# Supplementary material for: Regulation of CTCF loop formation during pancreatic cell differentiation
Source: Nat Commun. 2023 Oct 9;14:6314. doi: 10.1038/s41467-023-41964-6 (PMC10562423; doi:10.1038/s41467-023-41964-6)
Supplement: Supplementary file 1 — Supplementary Information [file 41467_2023_41964_MOESM1_ESM.pdf]

SUPPLEMENTARY INFORMATION

Regulation of CTCF loop formation during pancreatic cell differentiation

Xiaowen Lyu, M. Jordan Rowley, Michael J. Kulik, Stephen Dalton, and Victor G. Corces

Supplementary Figure 1

Supplementary Figure 2

Supplementary Figure 3

Supplementary Figure 4

Supplementary Figure 5

Supplementary Figure 6

Supplementary Figure 7

Supplementary Figure 8

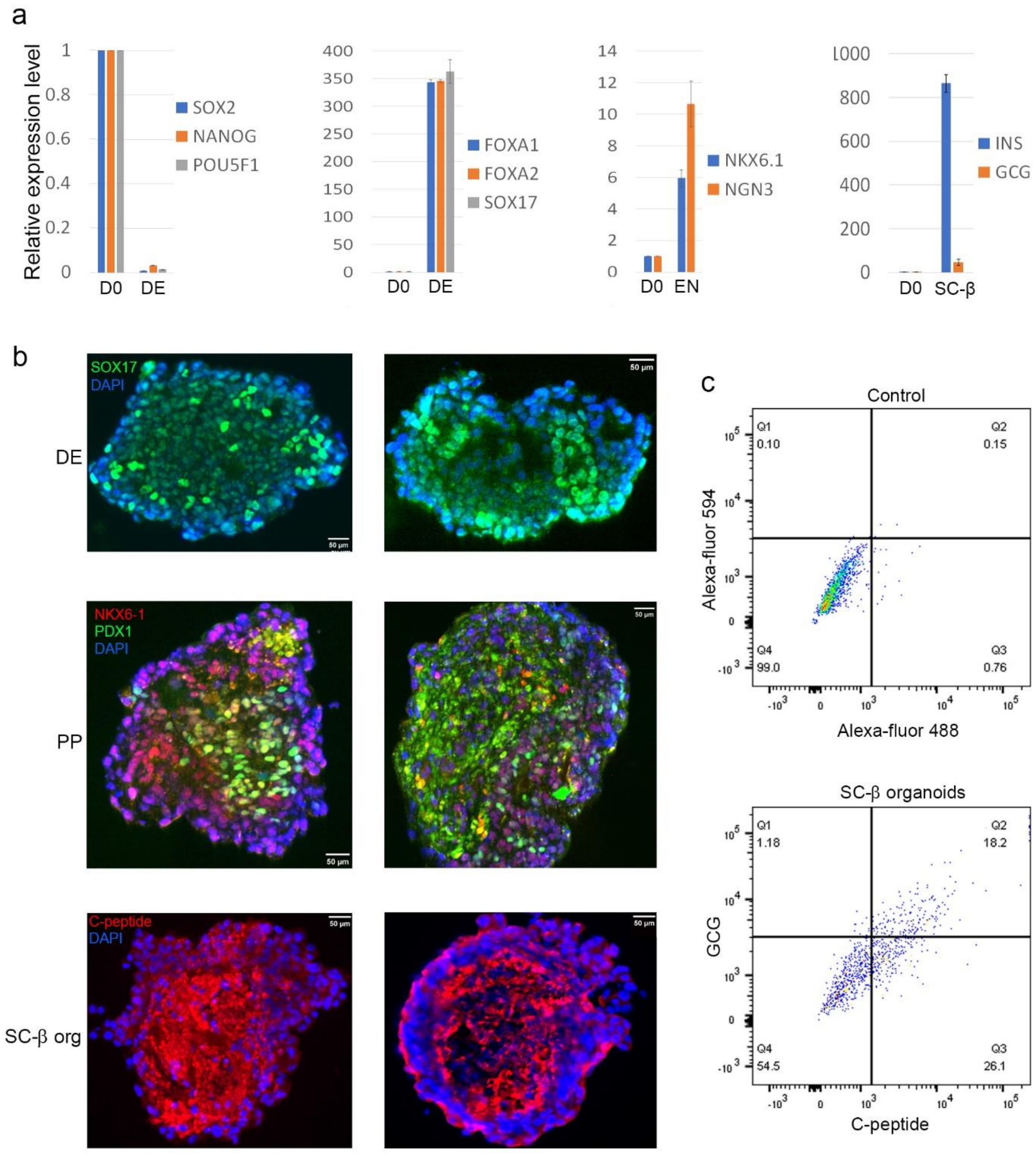

Supplementary Fig. 1 | *In vitro* directed differentiation of hESCs into pancreatic β cell organoids. a Validation of transcriptional changes of pluripotency marker genes and differentiation associated stage-specific marker genes by RT-qPCR. D0 indicates day 0 stem cell 3D spheres ready for differentiation; DE is definitive endoderm; EN is the intermediate endocrine progenitor stage between PP and SC-β organoids. **b** Evaluation of the efficiency of the differentiation process by measuring the percentages of cells expressing stage-specific marker genes by immunofluorescence histochemistry on cryosections of organoids. **c** FACS analysis quantifying the percentages of C-peptide +/GCG – cells in SC-β organoids.

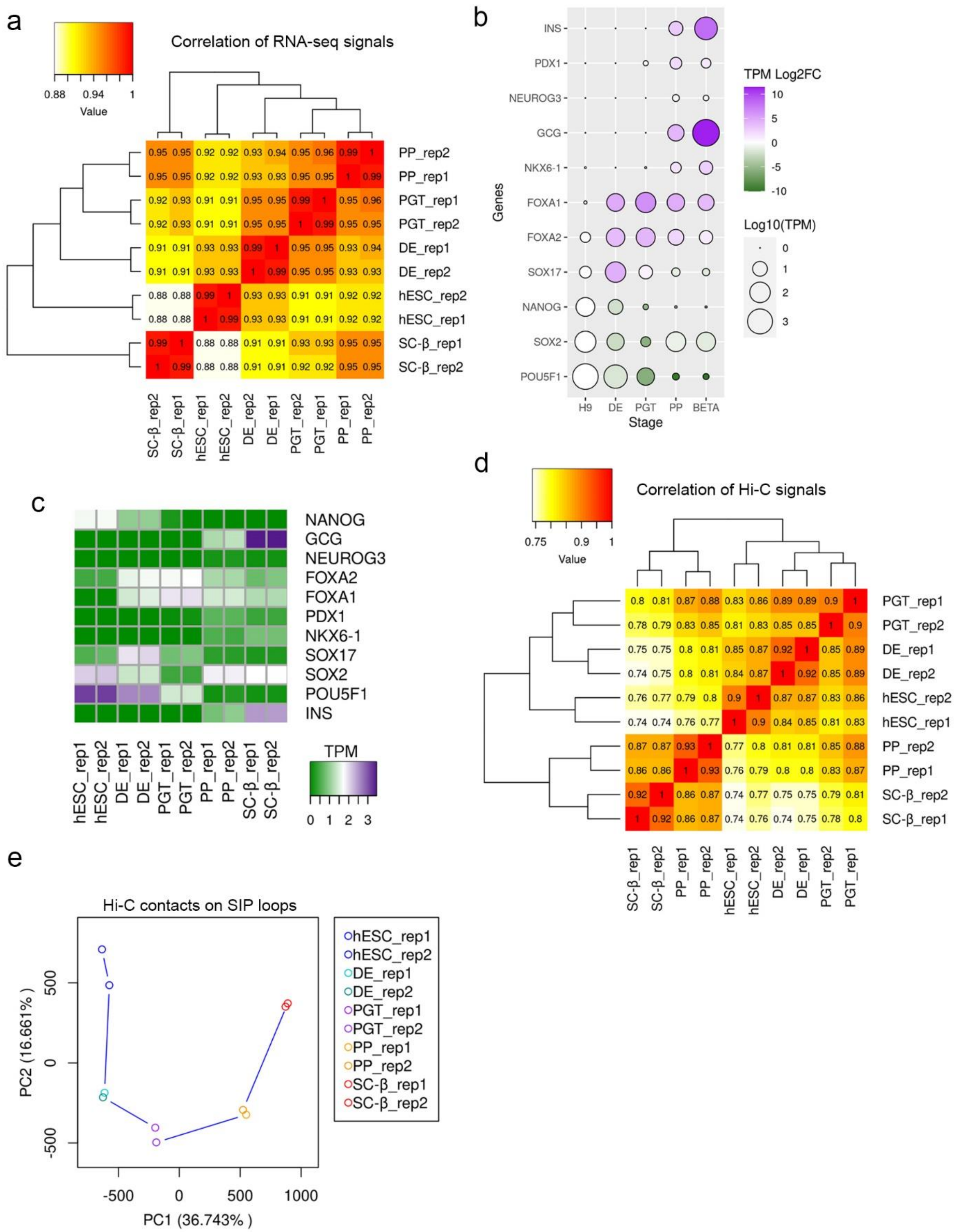

Supplementary Fig. 2 | Reproducibility of RNA-seq and Hi-C data between replicates at different stages of differentiation. a Correlation of RNA-seq signals between individual replicates. **b** Expression of marker genes for each differentiation stage based on RNA-seq data. **c** Correlation of marker gene expression between differentiation replicates. **d** Correlation of Hi-C data between independent replicates. **e** PCA analysis of Hi-C replicates.

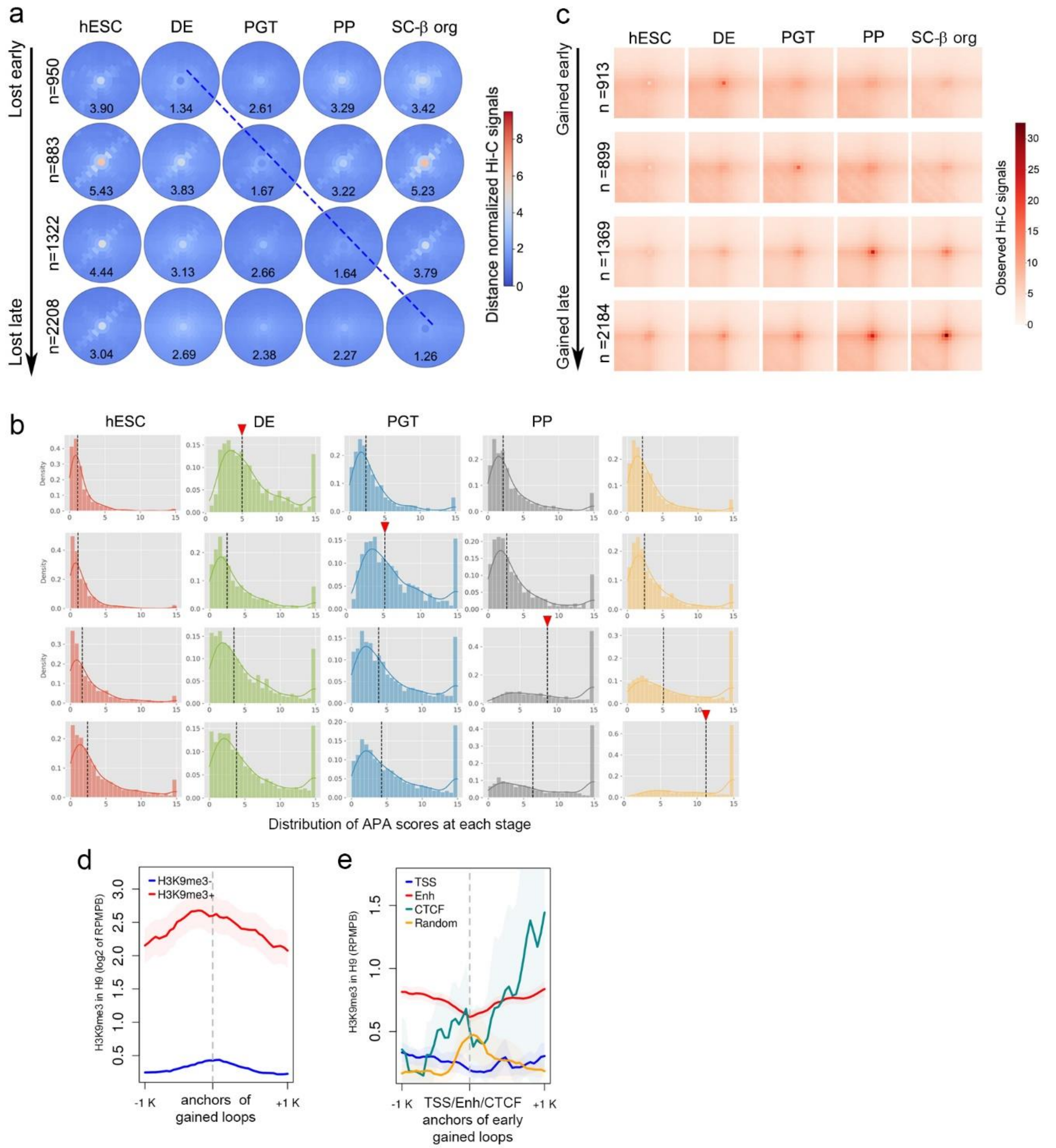

Supplementary Fig. 3 | Examination of aggregate peak analysis (APA) plots obtained using SIPMeta. a APA analyses of lost stage specific loops from Hi-C data obtained in cells at different stages during the differentiation of H9 hESCs into SC-β organoids. Each row shows CTCF loop APA values in cells at different stages for CTCF loops lost at each stage. For example, the top row shows APA values of CTCF loops present in H9 hESCs cells and lost in DE as well as the APA values of these CTCF loops at other stages of differentiation. **b** Histograms showing the distribution of fold changes of APA loop signal for all loop classes. **c** APA analysis of stage specific loops shown in Fig. 1a but using Hi-C data without distance normalization. **d** Average levels of H3K9me3 at CTCF loop anchors containing high or low levels of this histone modification. **e** Levels of H3K9me3 at CTCF loop anchors, adjacent enhancers and promoters, and random regions.

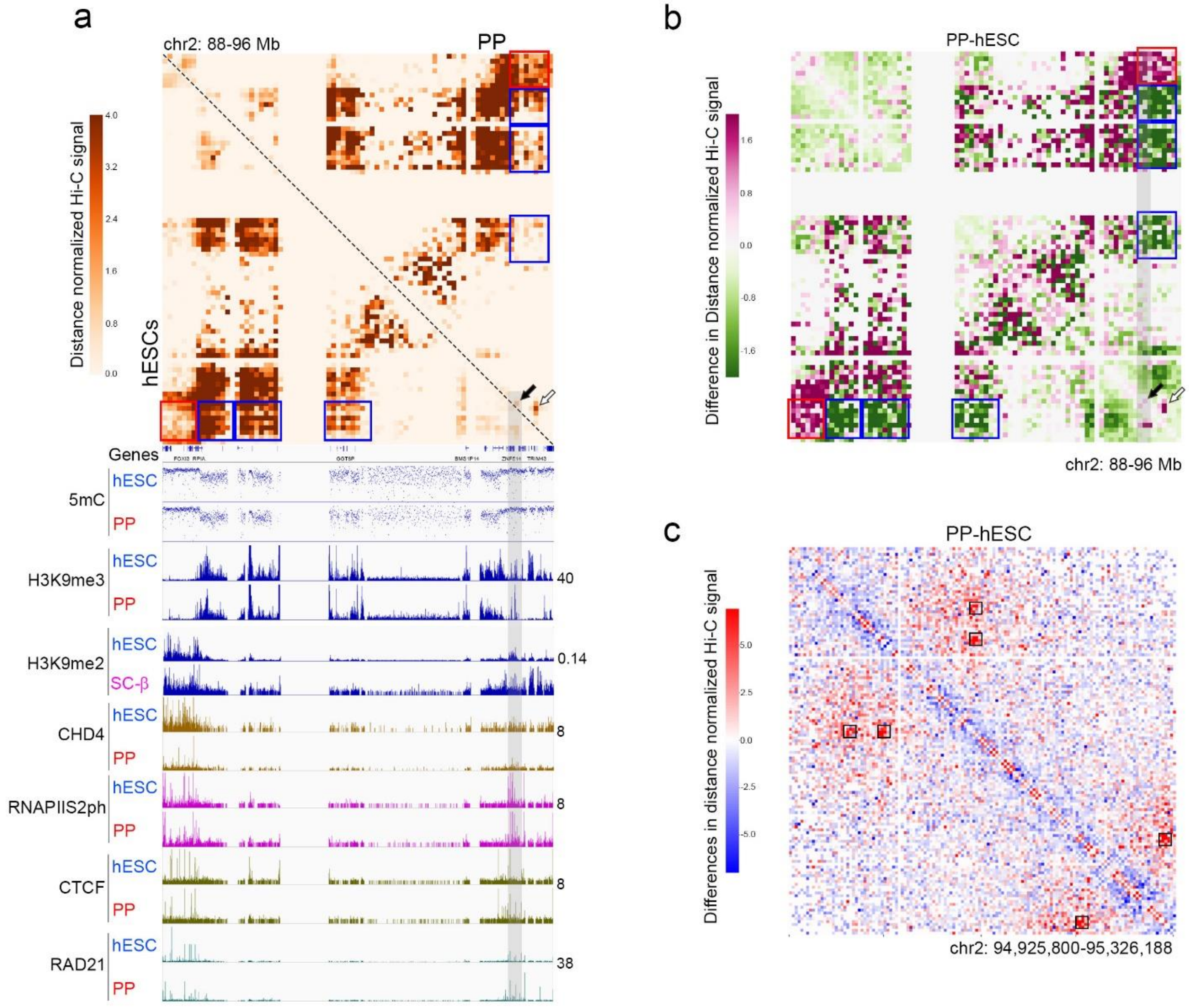

Supplementary Fig. 4 | Analysis of Hi-C data from cells at different stages during the differentiation from hESCs to SC-β organoids. a Changes in compartmental interactions (boxes) and CTCF loops (arrows) between PP cells and H9 hESCs. Distribution of several proteins, DNA methylation, and several histone modifications in PP and hESCs in the same region are also shown. **b** Subtraction heatmaps of Hi-C data showing gain (red squares) and loss (blue squares) of compartmental interactions between PP cells and H9 hESCs. **c** Subtraction Hi-C heatmap showing gain of CTCF loops (black squares) between PP cells and H9 hESCs.

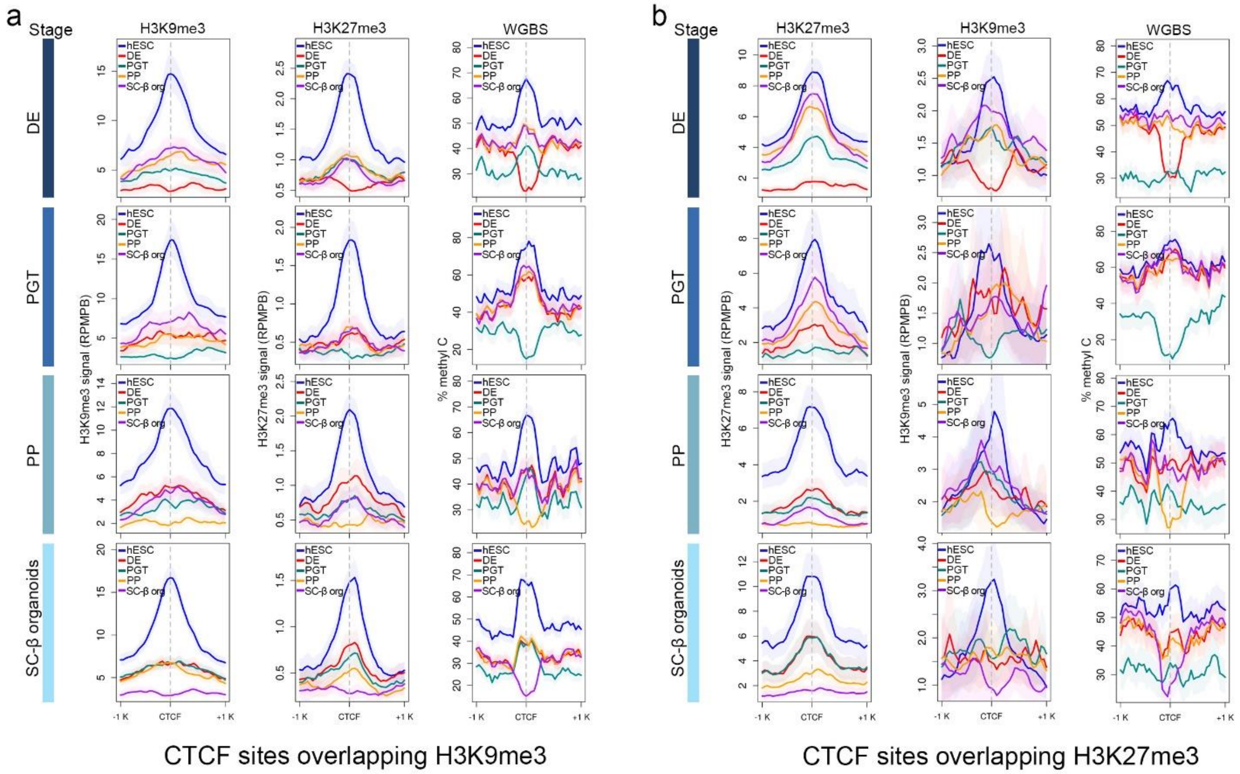

Supplementary Fig. 5 | Changes in histone modifications and DNA methylation at CTCF loop anchors. CTCF sites present at loop anchors were separated into those overlapping with peaks of H3K9me3 called by MACS and those overlapping H3K27me3 peaks. **a** CTC loop anchors overlapping with H3K9me3 peaks also contain approximately 10-fold lower amounts of H3K27me3. Loop anchors active in each stage contained high levels of both histone modifications in hESCs. For each stage, both histone modifications are at their lowest levels at their active anchors, which become populated by both histone modifications at all subsequent stages. **b** CTCF loop anchors that overlap with H3K27me3 peaks also contain slightly lower but similar levels of H3K9me3. Both modifications decrease at loop anchors active at each stage and increase again at subsequent stages. Changes in DNA methylation show a higher correlation with changes in H3K9me3.

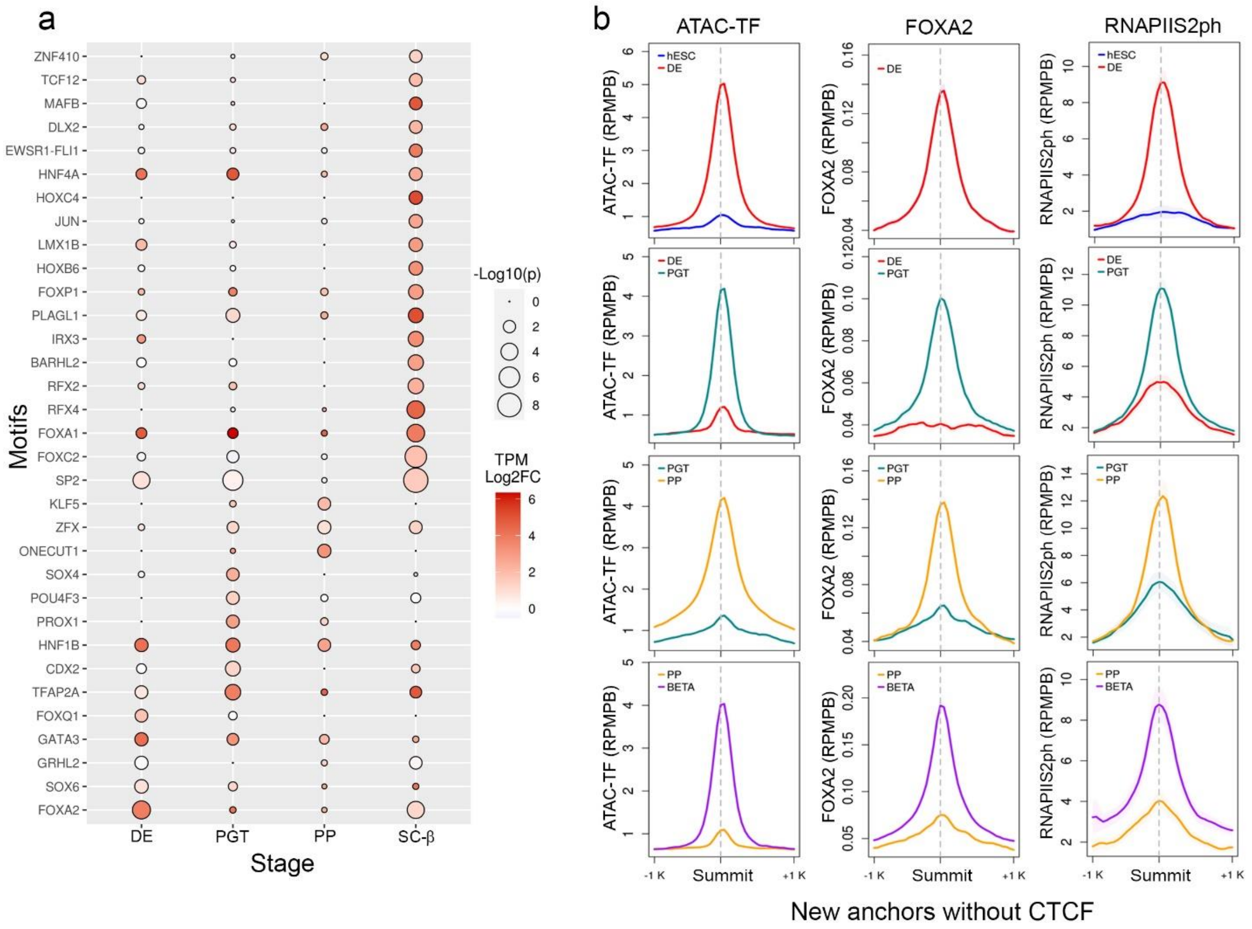

Supplementary Fig. 6 | Changes during pancreatic cell differentiation at new stage specific anchors lacking CTCF. a Analysis of TF binding motifs at the summits of ATAC-seq peaks present at 10 kb loop anchors lacking CTCF ChIP-seq peaks. **b** Changes in the distribution of chromatin accessibility, FOXA2, and RNAPII phosphorylated in Ser2 in ATAC-seq peaks located at loop anchors lacking CTCF.

a Enhancers and promoters at new anchors

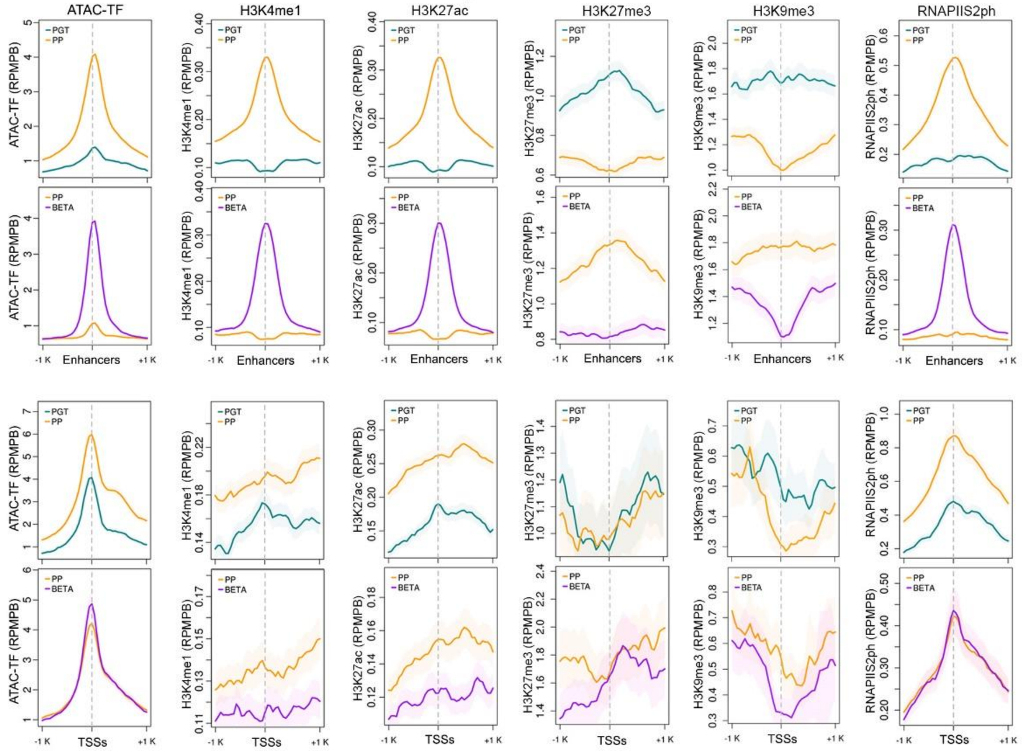

b Enhancers and promoters at old anchors

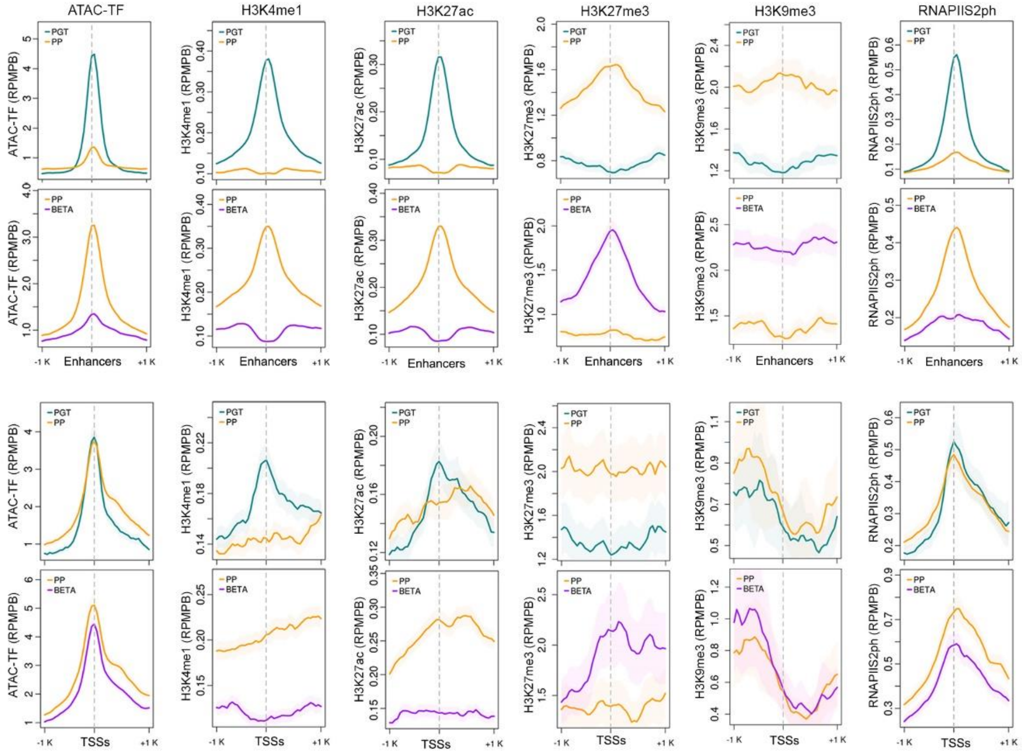

Supplementary Fig. 7 | Chromatin changes at enhancers and TSSs adjacent to new and old CTCF loop anchors. a Formation of CTCF loops by recruiting CTCF to new anchor sites correlates with an increase in ATAC-TF signal at adjacent enhancers and transcription start sites (TSSs). These enhancers also show increased H3K4me1, H3K27ac, and RNAPIIS2ph but decreased H3K9me3 and H3K27me3. Promoters show similar changes over broader regions. **b** Dissolution of CTCF loops by discarding previously used anchor sites. When this happens at a specific stage, enhancers and TSSs adjacent to discarded CTCF anchor sites loose ATAC-TF signal, H3K4me1, H3K27ac, and RNAPIIS2ph, while gaining H3K27me3 and H3K9me3, suggesting that loss of CTCF anchors correlates with inactivation of adjacent enhancers and promoters.

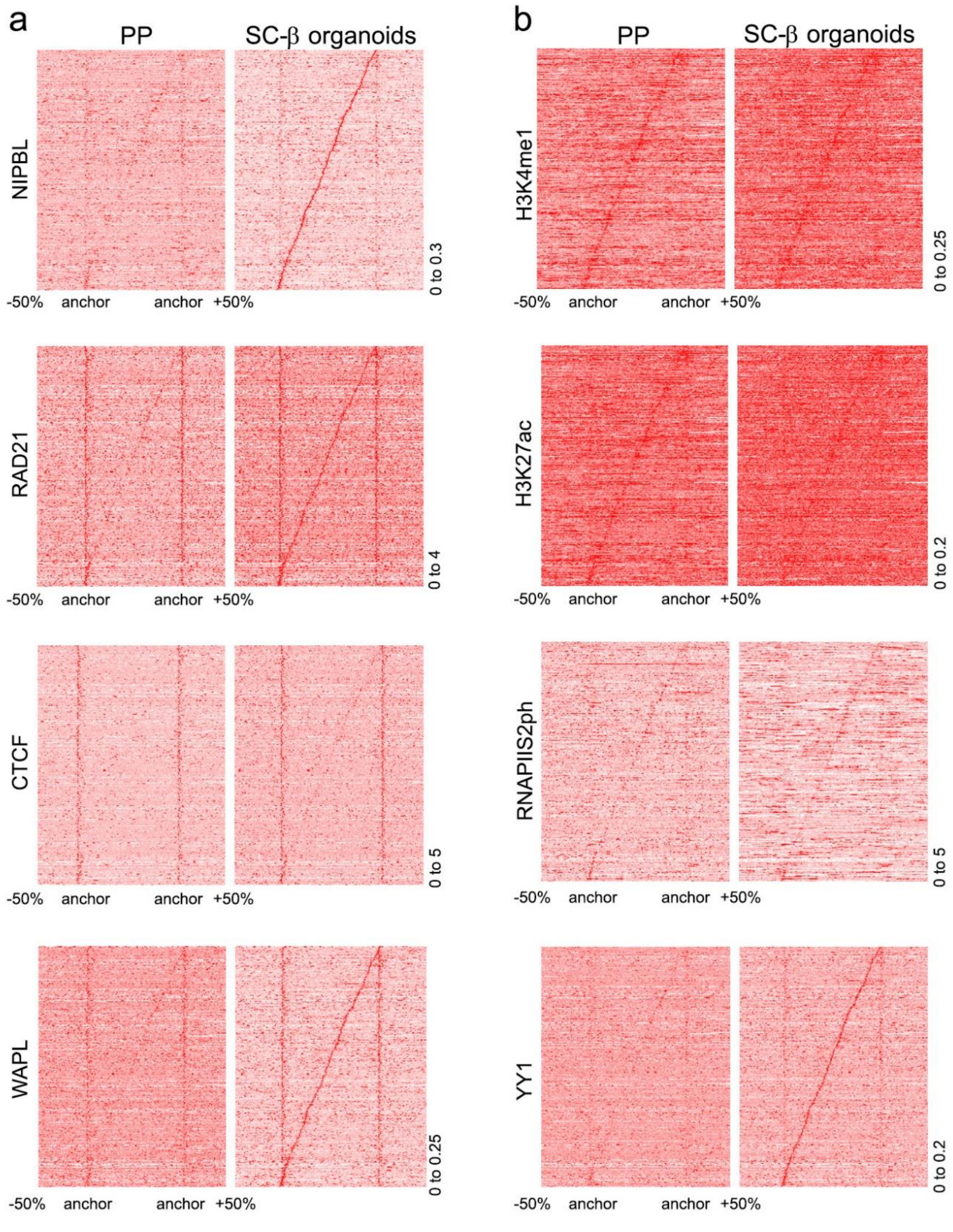

Supplementary Fig. 8 | Distribution of various proteins at CTCF loop anchors and cohesin loading sites. a Distribution of NIPBL, RAD21, CTCF and WAPL at loop anchors and loading sites of CTCF loops identified from Hi-C data present in SC-β organoids but not in PP cells. **b** Distribution of H3K4me1, H3K27ac, RNAPIIS2ph, and YY1 at loop anchors and loading sites of CTCF loops identified from Hi-C data present in SC-β organoids but not in PP cells.
