# Supplementary material for: Regulation of CTCF loop formation during pancreatic cell differentiation
Source: Nat Commun. 2023 Oct 9;14:6314. doi: 10.1038/s41467-023-41964-6 (PMC10562423; doi:10.1038/s41467-023-41964-6)
Supplement: Supplementary file 3 — Description of Additional Supplementary Files [file 41467_2023_41964_MOESM3_ESM.pdf]

Description of Additional Supplementary Files

File Name: Supplementary Data 1

Description: Quality control of Hi-C data. Each column indicates the name of each replicate for each of the samples corresponding to stages of differentiation of H9 hESCs into pancreatic cell progenitors, including definitive endoderm (DE), primitive gut tube-like (PGT), pancreatic progenitors (PP), and stem cell-derived β-cell organoids (SC-β organoids). Rows indicate the number of read pairs obtained at different steps of data processing for each sample replicate.
