# Supplementary material for: Regulation of CTCF loop formation during pancreatic cell differentiation
Source: Nat Commun. 2023 Oct 9;14:6314. doi: 10.1038/s41467-023-41964-6 (PMC10562423; doi:10.1038/s41467-023-41964-6)
Supplement: Supplementary file 5 — Reporting Summary [file 41467_2023_41964_MOESM5_ESM.pdf]

Corresponding author(s): Victor Corces
Last updated by author(s): 2023/09/19

Reporting Summary

Nature Portfolio wishes to improve the reproducibility of the work that we publish. This form provides structure for consistency and transparency in reporting. For further information on Nature Portfolio policies, see our Editorial Policies and the Editorial Policy Checklist.

Statistics

For all statistical analyses, confirm that the following items are present in the figure legend, table legend, main text, or Methods section.

n/a	Confirmed	
☐	☒	The exact sample size (*n*) for each experimental group/condition, given as a discrete number and unit of measurement
☐	☒	A statement on whether measurements were taken from distinct samples or whether the same sample was measured repeatedly
☐	☒	The statistical test(s) used AND whether they are one- or two-sided<br>*Only common tests should be described solely by name; describe more complex techniques in the Methods section.*
☒	☐	A description of all covariates tested
☒	☐	A description of any assumptions or corrections, such as tests of normality and adjustment for multiple comparisons
☐	☒	A full description of the statistical parameters including central tendency (e.g. means) or other basic estimates (e.g. regression coefficient) AND variation (e.g. standard deviation) or associated estimates of uncertainty (e.g. confidence intervals)
☐	☒	For null hypothesis testing, the test statistic (e.g. *F*, *t*, *r*) with confidence intervals, effect sizes, degrees of freedom and *P* value noted<br>*Give P values as exact values whenever suitable.*
☒	☐	For Bayesian analysis, information on the choice of priors and Markov chain Monte Carlo settings
☒	☐	For hierarchical and complex designs, identification of the appropriate level for tests and full reporting of outcomes
☐	☒	Estimates of effect sizes (e.g. Cohen's *d*, Pearson's *r*), indicating how they were calculated

Our web collection on statistics for biologists contains articles on many of the points above.

Software and code

Policy information about availability of computer code

Data collection

Description of all the commercial and open source analysis tools used in this study is listed in the Methods section of the paper

Data analysis

For statistical analyses, we used R (R studio version 1.3.1093),
For processing and analyzing next-gen sequencing data, we used the following tools and packages:
Juicer (Durand et al., 2016) https://github.com/theaidenlab/juicer/wiki
HiC-Pro (Servant, N., et al.， 2015) https://github.com/nservant/HiC-Pro
Juicebox (Durand et al., 2016) http://aidenlab.org/juicebox/
MACS2.0 (Liu, 2014)https://github.com/taoliu/MACS
Bowtie2 (Langmead, 2012) https://bowtie-bio.sourceforge.net/bowtie2/index.shtml
HISAT2 (Kim, D et al., 2019) https://github.com/DaehwanKimLab/hisat2
StringTie (Pertea, M et al., 2015) https://ccb.jhu.edu/software/stringtie/
Bismark (Krueger F, 2011) http://felixkrueger.github.io/Bismark/Docs/
SIP (Rowley MJ et al., 2020a) https://github.com/PouletAxel/SIP
SIPMeta (Rowley MJ et al., 2020b) https://github.com/PouletAxel/SIPMeta
Samtools (Li et al., 2009) https://sourceforge.net/projects/samtools/files/
Picard Tools http://picard.sourceforge.net; https://broadinstitute.github.io/picard/
TOPHAT2 (Kim et al., 2013) https://github.com/infphilo/tophat
Cufflinks (Trapnell et al., 2010) http://cole-trapnell-lab.github.io/cufflinks
DANPOS (Chen et al., 2013) https://sites.google.com/site/danposdoc/install
bedtools (Quinlan, 2014) http://bedtools.readthedocs.io/en/latest/
EdgeR （Robinson et al., 2010） https://bioconductor.org/packages/release/bioc/html/edgeR.html/

MAnorm (Shao et al., 2012) http://bcb.dfci.harvard.edu/~gcyuan/MAnorm/MAnorm.htm
Wellington-bootstrap (Piper et al., 2013) http://pythonhosted.org/pyDNase/
Java treeview https://sourceforge.net/projects/jtreeview/files/
Cluster3 http://bonsai.hgc.jp/~mdehoon/software/cluster/software.htm
FIMO (Grant et al., 2011) http://meme-suite.org/
MEME (Bailey et al., 2006) http://meme-suite.org/
FitHiChIP (Bhattacharyya, S al., 2019) https://ay-lab.github.io/FitHiChIP/
Fit-Hi-C (Ay et al., 2014) https://noble.gs.washington.edu/proj/fit-hi-c/
For imaging analysis: ImageJ(https://imagej.nih.gov/ij/)
For flow cytometry analysis: FlowJo(v 10.3) (https://www.flowjo.com/)

For manuscripts utilizing custom algorithms or software that are central to the research but not yet described in published literature, software must be made available to editors and reviewers. We strongly encourage code deposition in a community repository (e.g. GitHub). See the Nature Portfolio guidelines for submitting code & software for further information.

Data

Policy information about availability of data

All manuscripts must include a data availability statement. This statement should provide the following information, where applicable:

- Accession codes, unique identifiers, or web links for publicly available datasets
- A description of any restrictions on data availability
- For clinical datasets or third party data, please ensure that the statement adheres to our policy

All data sets generated in this study are deposited in the NCBI Gene Expression Omnibus (GEO; https://www.ncbi.nlm.nih.gov/geo/) under the following accession numbers. RNA-seq, ATAC-seq, ChIP-seq and CUT&Tag data are available under accession number GSE211101 (https://www.ncbi.nlm.nih.gov/geo/query/acc.cgi?acc=GSE211101). Hi-C data is available under accession number GSE210524 (https://www.ncbi.nlm.nih.gov/geo/query/acc.cgi?acc=GSE210524). HiChIP and MiChIP data are available under accession number GSE210525 (https://www.ncbi.nlm.nih.gov/geo/query/acc.cgi?acc=GSE210525). The various datasets reported in the manuscript can be visualized in the UCSC browser using the following link https://genome.ucsc.edu/cgi-bin/hgTracks?db=hg38&lastVirtModeType=default&lastVirtModeExtraState=&virtModeType=default&virtMode=0&nonVirtPosition=&position=chr2%3A25160915%2D25168903&hgsid=1711140546_4oiO7H0pztEcMSrKRlpnSCYhbDET.

Research involving human participants, their data, or biological material

Policy information about studies with human participants or human data. See also policy information about sex, gender (identity/presentation), and sexual orientation and race, ethnicity and racism.

Reporting on sex and gender	n/a
Reporting on race, ethnicity, or other socially relevant groupings	n/a
Population characteristics	n/a
Recruitment	n/a
Ethics oversight	n/a

Note that full information on the approval of the study protocol must also be provided in the manuscript.

Field-specific reporting

Please select the one below that is the best fit for your research. If you are not sure, read the appropriate sections before making your selection.

☒ Life sciences ☐ Behavioural & social sciences ☐ Ecological, evolutionary & environmental sciences

For a reference copy of the document with all sections, see nature.com/documents/nr-reporting-summary-flat.pdf

Life sciences study design

All studies must disclose on these points even when the disclosure is negative.

Sample size	We used sample sizes commonly accepted in the field for next-gen high-throughput genome-wide experiments. The number of cells used for each type of experiment is determined by the number of reads required for the analysis and/or the experimental conditions. For example, ATAC-seq experiments are normally performed using 50,000 cells to account for the standard concentration of Tn5 used in the assay (see refs. 83 and 84 in the manuscript). ChiP-seq experiments require around 500,000 cells whereas Micro-C and Hi-C experiments require 1-2 million cells (see ref. 91 in the manuscript). The use of two replicates is common practice in molecular biology and genomics, all quantitative data shown is an average of at least 2 independent experiments. See ENCODE recommendations at https://www.encodeproject.org/data-standards/

Data exclusions	No data were excluded from these analyses since all the data generated in this study passed quality control.
Replication	All experiments have at least two independent biological replicates. All attempts to replicate experiments were successful.
Randomization	The study involves analyses of various types of genomics data in cells from specific stages of pancreatic differentiation. Thus, randomization is not applicable to our study.
Blinding	Blinding is not applicable to our study, experiments and analyses were performed in parallel using standard procedures.

Reporting for specific materials, systems and methods

We require information from authors about some types of materials, experimental systems and methods used in many studies. Here, indicate whether each material, system or method listed is relevant to your study. If you are not sure if a list item applies to your research, read the appropriate section before selecting a response.

Materials & experimental systems

n/a	Involved in the study	
☐	☒	Antibodies
☐	☒	Eukaryotic cell lines
☒	☐	Palaeontology and archaeology
☒	☐	Animals and other organisms
☒	☐	Clinical data
☒	☐	Dual use research of concern
☒	☐	Plants

Methods

n/a	Involved in the study	
☐	☒	ChIP-seq
☐	☒	Flow cytometry
☒	☐	MRI-based neuroimaging

Antibodies

Antibodies used

Anti-RAD21 Abcam ab992
Anti-CTCF Abcam ab70303
Anti-YY1 Abcam ab109237
Anti-WAPL BETHYL A300-268A
Anti-NIPBL BETHYL A301-779A
Anti-cJun Abcam ab31419
Anti-RNAPII RPB1 Biolegend 920102
Anti-RNA polymerase II CTD repeat YSPTSPS (phospho S2) Abcam ab5095
Anti-SA1 BETHYL A302-579A
Anti-SA2 BETHYL A302-580A
Anti-CHD4 Abcam ab72418
Anti-FOXA2 Abcam ab256493
Anti-ESCO1 Sigma SAB1408225
Anti-H3K4me1 Abcam ab8895
Anti-H3K9me3 Abcam ab8898
Anti-H3K27ac Abcam ab4729
Anti-H3K9me2 Abcam ab1220
Anti-SOX17 R&D AF1924
Anti-PDX1 R&D AF2419
Anti-NKX6.1 DSHB F55A12
Anti-C-peptide DSHB GN-ID4
Anti-Glucagon R&D MAB1249-SP
Anti-HDAC8 Abcam ab190954
Chicken anti-Mouse IgG (H+L) Cross-Adsorbed Secondary Antibody, Alexa Fluor 594 Thermo A-21201
Goat Anti-Rat IgG H&L (Alexa Fluor® 488) Abcam ab150157
Donkey anti-Goat IgG (H+L) Cross-Adsorbed Secondary Antibody, Alexa Fluor 488 Thermo A-11055

Validation

All primary antibodies used in this study are commercially available and have been shown to react with the appropriate human proteins on the manufacturer's websites by Western blotting or other assays. All secondary antibodies used in this study are commercially available and have been validated by the manufacturer.

Eukaryotic cell lines

Policy information about cell lines and Sex and Gender in Research

Cell line source(s)	WA09 hESCs were obtained from Wicell (https://www.wicell.org/home/stem-cells/catalog-of-stem-cell-lines/wa09.cmsx)
Authentication	Hi-C karyotyping and morphology

Mycoplasma contamination	Negative
Commonly misidentified lines (See ICLAC register)	No commonly misidentified cell lines were used in this study.

Plants

Seed stocks	*Report on the source of all seed stocks or other plant material used. If applicable, state the seed stock centre and catalogue number. If plant specimens were collected from the field, describe the collection location, date and sampling procedures.*
Novel plant genotypes	*Describe the methods by which all novel plant genotypes were produced. This includes those generated by transgenic approaches, gene editing, chemical/radiation-based mutagenesis and hybridization. For transgenic lines, describe the transformation method, the number of independent lines analyzed and the generation upon which experiments were performed. For gene-edited lines, describe the editor used, the endogenous sequence targeted for editing, the targeting guide RNA sequence (if applicable) and how the editor was applied.*
Authentication	*Describe any authentication procedures for each seed stock used or novel genotype generated. Describe any experiments used to assess the effect of a mutation and, where applicable, how potential secondary effects (e.g. second site T-DNA insertions, mosiacism, off-target gene editing) were examined.*

ChIP-seq

Data deposition

☒ Confirm that both raw and final processed data have been deposited in a public database such as GEO.

☒ Confirm that you have deposited or provided access to graph files (e.g. BED files) for the called peaks.

Data access links<br>*May remain private before publication.*	To review GEO accession GSE211101:<br>Go to https://www.ncbi.nlm.nih.gov/geo/query/acc.cgi?acc=GSE211101<br>Enter token szcjioastbulrwn into the box<br>To review GEO accession GSE210525:<br>Go to https://www.ncbi.nlm.nih.gov/geo/query/acc.cgi?acc=GSE210525<br>Enter token ihkvysykttofbab into the box<br>To review GEO accession GSE210524:<br>Go to https://www.ncbi.nlm.nih.gov/geo/query/acc.cgi?acc=GSE210524<br>Enter token mdejyugitnknzgr into the box
Files in database submission	BETA_Rep1_RNASeq_R1.fastq.gz<br>BETA_Rep1_RNASeq_R2.fastq.gz<br>BETA_Rep2_RNASeq_R1.fastq.gz<br>BETA_Rep2_RNASeq_R2.fastq.gz<br>DE_Rep1_RNASeq_R1.fastq.gz<br>DE_Rep1_RNASeq_R2.fastq.gz<br>DE_Rep2_RNASeq_R1.fastq.gz<br>DE_Rep2_RNASeq_R2.fastq.gz<br>H9_Rep1_RNASeq_R1.fastq.gz<br>H9_Rep1_RNASeq_R2.fastq.gz<br>H9_Rep2_RNASeq_R1.fastq.gz<br>H9_Rep2_RNASeq_R2.fastq.gz<br>PGT_Rep1_RNASeq_R1.fastq.gz<br>PGT_Rep1_RNASeq_R2.fastq.gz<br>PGT_Rep2_RNASeq_R1.fastq.gz<br>PGT_Rep2_RNASeq_R2.fastq.gz<br>PH_Rep1_RNASeq_R1.fastq.gz<br>PH_Rep1_RNASeq_R2.fastq.gz<br>PH_Rep2_RNASeq_R1.fastq.gz<br>PH_Rep2_RNASeq_R2.fastq.gz<br>PP_Rep1_RNASeq_R1.fastq.gz<br>PP_Rep1_RNASeq_R2.fastq.gz<br>PP_Rep2_RNASeq_R1.fastq.gz<br>PP_Rep2_RNASeq_R2.fastq.gz<br>BETA_omniATAC_Rep1_2lanescomb_R1.fastq.gz<br>BETA_omniATAC_Rep1_2lanescomb_R2.fastq.gz<br>BETA_omniATAC_Rep2_2lanescomb_R1.fastq.gz<br>BETA_omniATAC_Rep2_2lanescomb_R2.fastq.gz<br>DE_omniATAC_Rep1_2lanescomb_R1.fastq.gz<br>PGT_DpnII_HiC_Rep1_R1.fastq.gz<br>PGT_DpnII_HiC_Rep1_R2.fastq.gz<br>PP_DpnII_HiC_Rep2_R1.fastq.gz<br>PP_DpnII_HiC_Rep2_R2.fastq.gz<br>H9_DpnII_HiC_Rep2_R1.fastq.gz<br>H9_DpnII_HiC_Rep2_R2.fastq.gz

DE_DpnII_HiC_Rep2_R1.fastq.gz
DE_DpnII_HiC_Rep2_R2.fastq.gz
DE_DpnII_HiC_Rep1_R1.fastq.gz
PP_DpnII_HiC_Rep1_R1.fastq.gz
DE_DpnII_HiC_Rep1_R2.fastq.gz
BETA_DpnII_HiC_Rep1_R1.fastq.gz
PP_DpnII_HiC_Rep1_R2.fastq.gz
PH_DpnII_HiC_Rep1_R1.fastq.gz
PH_DpnII_HiC_Rep1_R2.fastq.gz
BETA_DpnII_HiC_Rep1_R2.fastq.gz
PGT_DpnII_HiC_Rep2_R1.fastq.gz
PGT_DpnII_HiC_Rep2_R2.fastq.gz
PH_DpnII_HiC_Rep2_R1.fastq.gz
BETA_DpnII_HiC_Rep2_R2.fastq.gz
BETA_DpnII_HiC_Rep2_R1.fastq.gz
PH_DpnII_HiC_Rep2_R2.fastq.gz
H9_DpnII_HiC_Rep1_R1.fastq.gz
H9_DpnII_HiC_Rep1_R2.fastq.gz
DE_omniATAC_Rep1_2lanescomb_R2.fastq.gz
DE_omniATAC_Rep2_2lanescomb_R1.fastq.gz
DE_omniATAC_Rep2_2lanescomb_R2.fastq.gz
PGT_omniATAC_Rep1_2lanescomb_R1.fastq.gz
PGT_omniATAC_Rep1_2lanescomb_R2.fastq.gz
PGT_omniATAC_Rep2_2lanescomb_R1.fastq.gz
PGT_omniATAC_Rep2_2lanescomb_R2.fastq.gz
PP_omniATAC_Rep1_2lanescomb_R1.fastq.gz
PP_omniATAC_Rep1_2lanescomb_R2.fastq.gz
PP_omniATAC_Rep2_2lanescomb_R1.fastq.gz
PP_omniATAC_Rep2_2lanescomb_R2.fastq.gz
BETA_Chd4_live_Cuttag_Rep1_2lanescomb_R1.fastq.gz
BETA_Chd4_live_Cuttag_Rep1_2lanescomb_R2.fastq.gz
BETA_Chd4_live_Cuttag_Rep2_2lanescomb_R1.fastq.gz
BETA_Chd4_live_Cuttag_Rep2_2lanescomb_R2.fastq.gz
DE_Chd4_live_Cuttag_Rep1_2lanescomb_R1.fastq.gz
DE_Chd4_live_Cuttag_Rep1_2lanescomb_R2.fastq.gz
DE_Chd4_live_Cuttag_Rep2_2lanescomb_R1.fastq.gz
DE_Chd4_live_Cuttag_Rep2_2lanescomb_R2.fastq.gz
H9_Chd4_live_Cuttag_Rep1_2lanescomb_R1.fastq.gz
H9_Chd4_live_Cuttag_Rep1_2lanescomb_R2.fastq.gz
H9_Chd4_live_Cuttag_Rep2_2lanescomb_R1.fastq.gz
H9_Chd4_live_Cuttag_Rep2_2lanescomb_R2.fastq.gz
PGT_Chd4_live_Cuttag_Rep1_2lanescomb_R1.fastq.gz
PGT_Chd4_live_Cuttag_Rep1_2lanescomb_R2.fastq.gz
PGT_Chd4_live_Cuttag_Rep2_2lanescomb_R1.fastq.gz
PGT_Chd4_live_Cuttag_Rep2_2lanescomb_R2.fastq.gz
PH_Chd4_live_Cuttag_Rep1_2lanescomb_R1.fastq.gz
PH_Chd4_live_Cuttag_Rep1_2lanescomb_R2.fastq.gz
PH_Chd4_live_Cuttag_Rep2_2lanescomb_R1.fastq.gz
PH_Chd4_live_Cuttag_Rep2_2lanescomb_R2.fastq.gz
PP_Chd4_live_Cuttag_Rep1_2lanescomb_R1.fastq.gz
PP_Chd4_live_Cuttag_Rep1_2lanescomb_R2.fastq.gz
PP_Chd4_live_Cuttag_Rep2_2lanescomb_R1.fastq.gz
PP_Chd4_live_Cuttag_Rep2_2lanescomb_R2.fastq.gz
H9_ChIPmentation_Input_R2.fastq.gz
BETA_ChIPmentation_Input_R2.fastq.gz
BETA_ChIPmentation_Input_R1.fastq.gz
PGT_ChIPmentation_Input_R1.fastq.gz
PH_ChIPmentation_Input_R2.fastq.gz
PH_ChIPmentation_Input_R1.fastq.gz
PP_ChIPmentation_Input_R2.fastq.gz
PP_ChIPmentation_Input_R1.fastq.gz
PGT_ChIPmentation_Input_R2.fastq.gz
DE_ChIPmentation_Input_R2.fastq.gz
DE_ChIPmentation_Input_R1.fastq.gz
H9_ChIPmentation_Input_R1.fastq.gz
BETA_cJun_CPmnt.2lanescombed_R1.fastq.gz
BETA_cJun_CPmnt.2lanescombed_R2.fastq.gz
DE_cJun_CPmnt_Rep1_2lanescomb_R1.fastq.gz
DE_cJun_CPmnt_Rep1_2lanescomb_R2.fastq.gz
DE_cJun_CPmnt_Rep2_2lanescomb_R1.fastq.gz
DE_cJun_CPmnt_Rep2_2lanescomb_R2.fastq.gz
H9_cJun_CPmnt.2lanescombed_R1.fastq.gz
H9_cJun_CPmnt.2lanescombed_R2.fastq.gz
PH_cJun_CPmnt_Rep1_2lanescomb_R1.fastq.gz
PH_cJun_CPmnt_Rep1_2lanescomb_R2.fastq.gz
PH_cJun_CPmnt_Rep2_2lanescomb_R1.fastq.gz

PH_cJun_CPmnt_Rep2_2lanescomb_R2.fastq.gz
PH-CTCF-CPmnt-Rep2_2lanesComb_R1.fastq.gz
PH-CTCF-CPmnt-Rep2_2lanesComb_R2.fastq.gz
BETA-CTCF-CPmnt-Rep2_2lanesComb_R1.fastq.gz
BETA-CTCF-CPmnt-Rep2_2lanesComb_R2.fastq.gz
PP-CTCF-CPmnt-Rep2_2lanesComb_R1.fastq.gz
PP-CTCF-CPmnt-Rep2_2lanesComb_R2.fastq.gz
H9-CTCF-CPmnt-Rep2_2lanesComb_R2.fastq.gz
H9-CTCF-CPmnt-Rep2_2lanesComb_R1.fastq.gz
PGT-CTCF-CPmnt-Rep2_2lanesComb_R1.fastq.gz
PGT-CTCF-CPmnt-Rep2_2lanesComb_R2.fastq.gz
DE-CTCF-CPmnt-Rep2_2lanesComb_R1.fastq.gz
DE-CTCF-CPmnt-Rep2_2lanesComb_R2.fastq.gz
BETA_FOXA2_CPmnt_Rep1_2lanescomb_R1.fastq.gz
BETA_FOXA2_CPmnt_Rep1_2lanescomb_R2.fastq.gz
BETA_FOXA2_CPmnt_Rep2_2lanescomb_R1.fastq.gz
BETA_FOXA2_CPmnt_Rep2_2lanescomb_R2.fastq.gz
DE_FOXA2_CPmnt_Rep1_2lanescomb_R1.fastq.gz
DE_FOXA2_CPmnt_Rep1_2lanescomb_R2.fastq.gz
DE_FOXA2_CPmnt_Rep2_2lanescomb_R1.fastq.gz
DE_FOXA2_CPmnt_Rep2_2lanescomb_R2.fastq.gz
PGT_FOXA2_CPmnt_Rep1_2lanescomb_R1.fastq.gz
PGT_FOXA2_CPmnt_Rep1_2lanescomb_R2.fastq.gz
PGT-FOXA2-CPmnt-Rep2_S1_L001_R1_001.fastq.gz
PGT-FOXA2-CPmnt-Rep2_S1_L001_R2_001.fastq.gz
PGT-FOXA2-CPmnt-Rep2_S1_L002_R1_001.fastq.gz
PGT-FOXA2-CPmnt-Rep2_S1_L002_R2_001.fastq.gz
PH_FOXA2_CPmnt_Rep1_2lanescomb_R1.fastq.gz
PH_FOXA2_CPmnt_Rep1_2lanescomb_R2.fastq.gz
PH_FOXA2_CPmnt_Rep2_2lanescomb_R1.fastq.gz
PH_FOXA2_CPmnt_Rep2_2lanescomb_R2.fastq.gz
PP_FOXA2_CPmnt_Rep1_2lanescomb_R1.fastq.gz
PP_FOXA2_CPmnt_Rep1_2lanescomb_R2.fastq.gz
PP-FOXA2-CPmnt-Rep2_S2_L001_R1_001.fastq.gz
PP-FOXA2-CPmnt-Rep2_S2_L001_R2_001.fastq.gz
PP-FOXA2-CPmnt-Rep2_S2_L002_R1_001.fastq.gz
PP-FOXA2-CPmnt-Rep2_S2_L002_R2_001.fastq.gz
BETA_H3K9me2_CPmnt_Rep1_2lanescomb_R1.fastq.gz
BETA_H3K9me2_CPmnt_Rep1_2lanescomb_R2.fastq.gz
BETA_H3K9me2_CPmnt_Rep2_2lanescomb_R1.fastq.gz
BETA_H3K9me2_CPmnt_Rep2_2lanescomb_R2.fastq.gz
DE_H3K9me2_CPmnt_Rep1_2lanescomb_R1.fastq.gz
DE_H3K9me2_CPmnt_Rep1_2lanescomb_R2.fastq.gz
DE_H3K9me2_CPmnt_Rep2_2lanescomb_R1.fastq.gz
DE_H3K9me2_CPmnt_Rep2_2lanescomb_R2.fastq.gz
H9_H3K9me2_CPmnt_Rep1_2lanescomb_R1.fastq.gz
H9_H3K9me2_CPmnt_Rep1_2lanescomb_R2.fastq.gz
H9_H3K9me2_CPmnt_Rep2_2lanescomb_R1.fastq.gz
H9_H3K9me2_CPmnt_Rep2_2lanescomb_R2.fastq.gz
PH_H3K9me2_CPmnt_Rep1_2lanescomb_R1.fastq.gz
PH_H3K9me2_CPmnt_Rep1_2lanescomb_R2.fastq.gz
PH_H3K9me2_CPmnt_Rep2_2lanescomb_R1.fastq.gz
PH_H3K9me2_CPmnt_Rep2_2lanescomb_R2.fastq.gz
BETA-H3K9me3-CPmnt-Rep2_2lanesComb_R1.fastq.gz
BETA-H3K9me3-CPmnt-Rep2_2lanesComb_R2.fastq.gz
DE-H3K9me3-CPmnt-Rep2_2lanesComb_R1.fastq.gz
DE-H3K9me3-CPmnt-Rep2_2lanesComb_R2.fastq.gz
H9-H3K9me3-CPmnt-Rep2_2lanesComb_R1.fastq.gz
H9-H3K9me3-CPmnt-Rep2_2lanesComb_R2.fastq.gz
PGT-H3K9me3-CPmnt-Rep2_2lanesComb_R1.fastq.gz
PGT-H3K9me3-CPmnt-Rep2_2lanesComb_R2.fastq.gz
PH-H3K9me3-CPmnt-Rep2_2lanesComb_R1.fastq.gz
PH-H3K9me3-CPmnt-Rep2_2lanesComb_R2.fastq.gz
PP-H3K9me3-CPmnt-Rep2_2lanesComb_R1.fastq.gz
PP-H3K9me3-CPmnt-Rep2_2lanesComb_R2.fastq.gz
BETA_H3K9me3_ChIPmentation_R1.fastq.gz
BETA_H3K9me3_ChIPmentation_R2.fastq.gz
DE_H3K9me3_ChIPmentation_R1.fastq.gz
DE_H3K9me3_ChIPmentation_R2.fastq.gz
H9_H3K9me3_ChIPmentation_R1.fastq.gz
H9_H3K9me3_ChIPmentation_R2.fastq.gz
PGT_H3K9me3_ChIPmentation_R1.fastq.gz
PGT_H3K9me3_ChIPmentation_R2.fastq.gz
PH_H3K9me3_ChIPmentation_R1.fastq.gz
PH_H3K9me3_ChIPmentation_R2.fastq.gz
PP_H3K9me3_ChIPmentation_R1.fastq.gz

PP_H3K9me3_ChIPmentation_R2.fastq.gz
BETA_NIPBL_CPmnt_Rep1_2lanescomb_R1.fastq.gz
BETA_NIPBL_CPmnt_Rep1_2lanescomb_R2.fastq.gz
BETA_NIPBL_CPmnt_Rep2_2lanescomb_R1.fastq.gz
BETA_NIPBL_CPmnt_Rep2_2lanescomb_R2.fastq.gz
DE_NIPBL_CPmnt_Rep1_2lanescomb_R1.fastq.gz
DE_NIPBL_CPmnt_Rep1_2lanescomb_R2.fastq.gz
DE_NIPBL_CPmnt_Rep2_2lanescomb_R1.fastq.gz
DE_NIPBL_CPmnt_Rep2_2lanescomb_R2.fastq.gz
H9_NIPBL_CPmnt_Rep1_2lanescomb_R1.fastq.gz
H9_NIPBL_CPmnt_Rep1_2lanescomb_R2.fastq.gz
H9_NIPBL_CPmnt_Rep2_2lanescomb_R1.fastq.gz
H9_NIPBL_CPmnt_Rep2_2lanescomb_R2.fastq.gz
PGT_NIPBL_CPmnt_Rep1_2lanescomb_R1.fastq.gz
PGT_NIPBL_CPmnt_Rep1_2lanescomb_R2.fastq.gz
PH_NIPBL_CPmnt_Rep1_2lanescomb_R1.fastq.gz
PH_NIPBL_CPmnt_Rep1_2lanescomb_R2.fastq.gz
PH_NIPBL_CPmnt_Rep2_2lanescomb_R1.fastq.gz
PH_NIPBL_CPmnt_Rep2_2lanescomb_R2.fastq.gz
PP_NIPBL_CPmnt_Rep1_2lanescomb_R1.fastq.gz
PP_NIPBL_CPmnt_Rep1_2lanescomb_R2.fastq.gz
BETA-Rad21-CPmnt-Rep2_2lanesComb_R1.fastq.gz
BETA-Rad21-CPmnt-Rep2_2lanesComb_R2.fastq.gz
DE-Rad21-CPmnt-Rep2_2lanesComb_R1.fastq.gz
DE-Rad21-CPmnt-Rep2_2lanesComb_R2.fastq.gz
H9-Rad21-CPmnt-Rep2_2lanesComb_R1.fastq.gz
H9-Rad21-CPmnt-Rep2_2lanesComb_R2.fastq.gz
PGT-Rad21-CPmnt-Rep2_2lanesComb_R1.fastq.gz
PGT-Rad21-CPmnt-Rep2_2lanesComb_R2.fastq.gz
PH-Rad21-CPmnt-Rep2_2lanesComb_R1.fastq.gz
PH-Rad21-CPmnt-Rep2_2lanesComb_R2.fastq.gz
PP-Rad21-CPmnt-Rep2_2lanesComb_R1.fastq.gz
PP-Rad21-CPmnt-Rep2_2lanesComb_R2.fastq.gz
DE_CTCF_ChIP_Rep_R1.rightidx.fastq.gz
DE_CTCF_ChIP_Rep_R2.rightidx.fastq.gz
H9_CTCF_ChIP_Rep_R1.rightidx.fastq.gz
H9_CTCF_ChIP_Rep_R2.rightidx.fastq.gz
PGT_CTCF_ChIP_Rep_R1.rightidx.fastq.gz
PGT_CTCF_ChIP_Rep_R2.rightidx.fastq.gz
PP_CTCF_ChIPmentation_R1.fastq.gz
PP_CTCF_ChIPmentation_R2.fastq.gz
BETA-S2P-CPmnt-Rep2_2lanesComb_R1.fastq.gz
BETA-S2P-CPmnt-Rep2_2lanesComb_R2.fastq.gz
DE-S2P-CPmnt-Rep2_2lanesComb_R1.fastq.gz
DE-S2P-CPmnt-Rep2_2lanesComb_R2.fastq.gz
H9-S2P-CPmnt-Rep2_2lanesComb_R1.fastq.gz
H9-S2P-CPmnt-Rep2_2lanesComb_R2.fastq.gz
PGT-S2P-CPmnt-Rep2_2lanesComb_R1.fastq.gz
PGT-S2P-CPmnt-Rep2_2lanesComb_R2.fastq.gz
PH-S2P-CPmnt-Rep2_2lanesComb_R1.fastq.gz
PH-S2P-CPmnt-Rep2_2lanesComb_R2.fastq.gz
PP-S2P-CPmnt-Rep2_2lanesComb_R1.fastq.gz
PP-S2P-CPmnt-Rep2_2lanesComb_R2.fastq.gz
BETA_S2P_ChIP_Rep_R1.rightidx.fastq.gz
BETA_S2P_ChIP_Rep_R2.rightidx.fastq.gz
PH_S2P_ChIP_Rep_R1.rightidx.fastq.gz
PH_S2P_ChIP_Rep_R2.rightidx.fastq.gz
BETA_WAPL_CPmnt_Rep1_2lanescomb_R1.fastq.gz
BETA_WAPL_CPmnt_Rep1_2lanescomb_R2.fastq.gz
BETA_WAPL_CPmnt_Rep2_2lanescomb_R1.fastq.gz
BETA_WAPL_CPmnt_Rep2_2lanescomb_R2.fastq.gz
DE_WAPL_CPmnt_Rep1_2lanescomb_R1.fastq.gz
DE_WAPL_CPmnt_Rep1_2lanescomb_R2.fastq.gz
DE_WAPL_CPmnt_Rep2_2lanescomb_R1.fastq.gz
DE_WAPL_CPmnt_Rep2_2lanescomb_R2.fastq.gz
H9_WAPL_CPmnt_Rep1_2lanescomb_R1.fastq.gz
H9_WAPL_CPmnt_Rep1_2lanescomb_R2.fastq.gz
H9_WAPL_CPmnt_Rep2_2lanescomb_R1.fastq.gz
H9_WAPL_CPmnt_Rep2_2lanescomb_R2.fastq.gz
PGT_WAPL_CPmnt_Rep1_2lanescomb_R1.fastq.gz
PGT_WAPL_CPmnt_Rep1_2lanescomb_R2.fastq.gz
PH_WAPL_CPmnt_Rep1_2lanescomb_R1.fastq.gz
PH_WAPL_CPmnt_Rep1_2lanescomb_R2.fastq.gz
PH_WAPL_CPmnt_Rep2_2lanescomb_R1.fastq.gz
PH_WAPL_CPmnt_Rep2_2lanescomb_R2.fastq.gz
PP_WAPL_CPmnt_Rep1_2lanescomb_R1.fastq.gz

PP_WAPL_CPmnt_Rep1_2lanescomb_R2.fastq.gz
BETA_YY1_CPmnt_Rep1_2lanescomb_R1.fastq.gz
BETA_YY1_CPmnt_Rep1_2lanescomb_R2.fastq.gz
BETA_YY1_CPmnt_Rep2_2lanescomb_R1.fastq.gz
BETA_YY1_CPmnt_Rep2_2lanescomb_R2.fastq.gz
DE_YY1_CPmnt_Rep1_2lanescomb_R1.fastq.gz
DE_YY1_CPmnt_Rep1_2lanescomb_R2.fastq.gz
DE_YY1_CPmnt_Rep2_2lanescomb_R1.fastq.gz
DE_YY1_CPmnt_Rep2_2lanescomb_R2.fastq.gz
H9_YY1_CPmnt_Rep1_2lanescomb_R1.fastq.gz
H9_YY1_CPmnt_Rep1_2lanescomb_R2.fastq.gz
H9_YY1_CPmnt_Rep2_2lanescomb_R1.fastq.gz
H9_YY1_CPmnt_Rep2_2lanescomb_R2.fastq.gz
PGT_YY1_CPmnt_Rep1_2lanescomb_R1.fastq.gz
PGT_YY1_CPmnt_Rep1_2lanescomb_R2.fastq.gz
PH_YY1_CPmnt_Rep1_2lanescomb_R1.fastq.gz
PH_YY1_CPmnt_Rep1_2lanescomb_R2.fastq.gz
PH_YY1_CPmnt_Rep2_2lanescomb_R1.fastq.gz
PH_YY1_CPmnt_Rep2_2lanescomb_R2.fastq.gz
PP_YY1_CPmnt_Rep1_2lanescomb_R1.fastq.gz
PP_YY1_CPmnt_Rep1_2lanescomb_R2.fastq.gz
PP_ESCO1_CPmnt_Rep2_2lanescomb_R2.fastq.gz
PP_ESCO1_CPmnt_Rep2_2lanescomb_R1.fastq.gz
PP_ESCO1_CPmnt_Rep1_2lanescomb_R2.fastq.gz
PP_ESCO1_CPmnt_Rep1_2lanescomb_R1.fastq.gz
PGT_ESCO1_CPmnt_Rep2_2lanescomb_R2.fastq.gz
PGT_ESCO1_CPmnt_Rep2_2lanescomb_R1.fastq.gz
PGT_ESCO1_CPmnt_Rep1_2lanescomb_R2.fastq.gz
PGT_ESCO1_CPmnt_Rep1_2lanescomb_R1.fastq.gz
H9_ESCO1_CPmnt_Rep2_2lanescomb_R2.fastq.gz
H9_ESCO1_CPmnt_Rep2_2lanescomb_R1.fastq.gz
H9_ESCO1_CPmnt_Rep1_2lanescomb_R2.fastq.gz
H9_ESCO1_CPmnt_Rep1_2lanescomb_R1.fastq.gz
DE_ESCO1_CPmnt_Rep2_2lanescomb_R2.fastq.gz
DE_ESCO1_CPmnt_Rep2_2lanescomb_R1.fastq.gz
DE_ESCO1_CPmnt_Rep1_2lanescomb_R2.fastq.gz
DE_ESCO1_CPmnt_Rep1_2lanescomb_R1.fastq.gz
BETA_ESCO1_CPmnt_Rep2_2lanescomb_R2.fastq.gz
BETA_ESCO1_CPmnt_Rep2_2lanescomb_R1.fastq.gz
BETA_ESCO1_CPmnt_Rep1_2lanescomb_R2.fastq.gz
BETA_ESCO1_CPmnt_Rep1_2lanescomb_R1.fastq.gz
PP_HDAC8_CPmnt_Rep2_2lanescomb_R2.fastq.gz
PP_HDAC8_CPmnt_Rep2_2lanescomb_R1.fastq.gz
PP_HDAC8_CPmnt_Rep1_2lanescomb_R2.fastq.gz
PP_HDAC8_CPmnt_Rep1_2lanescomb_R1.fastq.gz
PGT_HDAC8_CPmnt_Rep2_2lanescomb_R2.fastq.gz
PGT_HDAC8_CPmnt_Rep2_2lanescomb_R1.fastq.gz
PGT_HDAC8_CPmnt_Rep1_2lanescomb_R2.fastq.gz
PGT_HDAC8_CPmnt_Rep1_2lanescomb_R1.fastq.gz
H9_HDAC8_CPmnt_Rep2_2lanescomb_R2.fastq.gz
H9_HDAC8_CPmnt_Rep2_2lanescomb_R1.fastq.gz
H9_HDAC8_CPmnt_Rep1_2lanescomb_R2.fastq.gz
H9_HDAC8_CPmnt_Rep1_2lanescomb_R1.fastq.gz
DE_HDAC8_CPmnt_Rep2_2lanescomb_R2.fastq.gz
DE_HDAC8_CPmnt_Rep2_2lanescomb_R1.fastq.gz
DE_HDAC8_CPmnt_Rep1_2lanescomb_R2.fastq.gz
DE_HDAC8_CPmnt_Rep1_2lanescomb_R1.fastq.gz
BETA_HDAC8_CPmnt_Rep2_2lanescomb_R2.fastq.gz
BETA_HDAC8_CPmnt_Rep2_2lanescomb_R1.fastq.gz
BETA_HDAC8_CPmnt_Rep1_2lanescomb_R2.fastq.gz
BETA_HDAC8_CPmnt_Rep1_2lanescomb_R1.fastq.gz
BETA_H3K27ac_HiCPmnt_Rep1_2lanescomb_R1.fastq.gz
BETA_H3K27ac_HiCPmnt_Rep1_2lanescomb_R2.fastq.gz
BETA_H3K27ac_HiCPmnt_Rep2_2lanescomb_R1.fastq.gz
BETA_H3K27ac_HiCPmnt_Rep2_2lanescomb_R2.fastq.gz
DE_H3K27ac_HiCPmnt_Rep1_2lanescomb_R1.fastq.gz
DE_H3K27ac_HiCPmnt_Rep1_2lanescomb_R2.fastq.gz
H9_H3K27ac_HiCPmnt_Rep1_2lanescomb_R1.fastq.gz
H9_H3K27ac_HiCPmnt_Rep1_2lanescomb_R2.fastq.gz
H9_H3K27ac_HiCPmnt_Rep2_2lanescomb_R1.fastq.gz
H9_H3K27ac_HiCPmnt_Rep2_2lanescomb_R2.fastq.gz
PGT_H3K27ac_HiCPmnt_Rep1_2lanescomb_R1.fastq.gz
PGT_H3K27ac_HiCPmnt_Rep1_2lanescomb_R2.fastq.gz
PGT_H3K27ac_HiCPmnt_Rep2_2lanescomb_R1.fastq.gz
PGT_H3K27ac_HiCPmnt_Rep2_2lanescomb_R2.fastq.gz
PH_H3K27ac_HiCPmnt_Rep1_2lanescomb_R1.fastq.gz

PH_H3K27ac_HiCPmnt_Rep1_2lanescomb_R2.fastq.gz
PH_H3K27ac_HiCPmnt_Rep2_2lanescomb_R1.fastq.gz
PH_H3K27ac_HiCPmnt_Rep2_2lanescomb_R2.fastq.gz
PP_H3K27ac_HiCPmnt_Rep1_2lanescomb_R1.fastq.gz
PP_H3K27ac_HiCPmnt_Rep1_2lanescomb_R2.fastq.gz
PP_H3K27ac_HiCPmnt_Rep2_2lanescomb_R1.fastq.gz
PP_H3K27ac_HiCPmnt_Rep2_2lanescomb_R2.fastq.gz
BETA_H3K4me1_HiCPmnt_Rep1_2lanescomb_R1.fastq.gz
BETA_H3K4me1_HiCPmnt_Rep1_2lanescomb_R2.fastq.gz
BETA_H3K4me1_HiCPmnt_Rep2_2lanescomb_R1.fastq.gz
BETA_H3K4me1_HiCPmnt_Rep2_2lanescomb_R2.fastq.gz
DE_H3K4me1_HiCPmnt_Rep1_2lanescomb_R1.fastq.gz
DE_H3K4me1_HiCPmnt_Rep1_2lanescomb_R2.fastq.gz
H9_H3K4me1_HiCPmnt_Rep1_2lanescomb_R1.fastq.gz
H9_H3K4me1_HiCPmnt_Rep1_2lanescomb_R2.fastq.gz
H9_H3K4me1_HiCPmnt_Rep2_2lanescomb_R1.fastq.gz
H9_H3K4me1_HiCPmnt_Rep2_2lanescomb_R2.fastq.gz
PGT_H3K4me1_HiCPmnt_Rep1_2lanescomb_R1.fastq.gz
PGT_H3K4me1_HiCPmnt_Rep1_2lanescomb_R2.fastq.gz
PGT_H3K4me1_HiCPmnt_Rep2_2lanescomb_R1.fastq.gz
PGT_H3K4me1_HiCPmnt_Rep2_2lanescomb_R2.fastq.gz
PH_H3K4me1_HiCPmnt_Rep1_2lanescomb_R1.fastq.gz
PH_H3K4me1_HiCPmnt_Rep1_2lanescomb_R2.fastq.gz
PH_H3K4me1_HiCPmnt_Rep2_2lanescomb_R1.fastq.gz
PH_H3K4me1_HiCPmnt_Rep2_2lanescomb_R2.fastq.gz
PP_H3K4me1_HiCPmnt_Rep1_2lanescomb_R1.fastq.gz
PP_H3K4me1_HiCPmnt_Rep1_2lanescomb_R2.fastq.gz
PP_H3K4me1_HiCPmnt_Rep2_2lanescomb_R1.fastq.gz
PP_H3K4me1_HiCPmnt_Rep2_2lanescomb_R2.fastq.gz
BETA_H3K9me3_HiCPmnt_Rep1_R1.fastq.gz
BETA_H3K9me3_HiCPmnt_Rep1_R2.fastq.gz
BETA_H3K9me3_HiCPmnt_Rep2_R1.fastq.gz
BETA_H3K9me3_HiCPmnt_Rep2_R2.fastq.gz
DE_H3K9me3_HiCPmnt_Rep1_R1.fastq.gz
DE_H3K9me3_HiCPmnt_Rep1_R2.fastq.gz
DE_H3K9me3_HiCPmnt_Rep2_R1.fastq.gz
DE_H3K9me3_HiCPmnt_Rep2_R2.fastq.gz
H9_H3K9me3_HiCPmnt_Rep1_R1.fastq.gz
H9_H3K9me3_HiCPmnt_Rep1_R2.fastq.gz
H9_H3K9me3_HiCPmnt_Rep2_R1.fastq.gz
H9_H3K9me3_HiCPmnt_Rep2_R2.fastq.gz
PGT_H3K9me3_HiCPmnt_Rep1_R1.fastq.gz
PGT_H3K9me3_HiCPmnt_Rep1_R2.fastq.gz
PGT_H3K9me3_HiCPmnt_Rep2_R1.fastq.gz
PGT_H3K9me3_HiCPmnt_Rep2_R2.fastq.gz
PH_H3K9me3_HiCPmnt_Rep1_R1.fastq.gz
PH_H3K9me3_HiCPmnt_Rep1_R2.fastq.gz
PH_H3K9me3_HiCPmnt_Rep2_R1.fastq.gz
PH_H3K9me3_HiCPmnt_Rep2_R2.fastq.gz
PP_H3K9me3_HiCPmnt_Rep1_R1.fastq.gz
PP_H3K9me3_HiCPmnt_Rep1_R2.fastq.gz
PP_H3K9me3_HiCPmnt_Rep2_R1.fastq.gz
PP_H3K9me3_HiCPmnt_Rep2_R2.fastq.gz
BETA_PolII_HiCPmnt_Rep1_2Novaseqcomb_R1.fastq.gz
BETA_PolII_HiCPmnt_Rep1_2Novaseqcomb_R2.fastq.gz
BETA_PolII_HiCPmnt_Rep2_2Novaseqcomb_R1.fastq.gz
BETA_PolII_HiCPmnt_Rep2_2Novaseqcomb_R2.fastq.gz
DE_PolII_HiCPmnt_Rep1_2lanescomb_R1.fastq.gz
DE_PolII_HiCPmnt_Rep1_2lanescomb_R2.fastq.gz
H9_PolII_HiCPmnt_Rep1_2Novaseqcomb_R1.fastq.gz
H9_PolII_HiCPmnt_Rep1_2Novaseqcomb_R2.fastq.gz
H9_PolII_HiCPmnt_Rep2_2Novaseqcomb_R1.fastq.gz
H9_PolII_HiCPmnt_Rep2_2Novaseqcomb_R2.fastq.gz
PGT_PolII_HiCPmnt_Rep1_2Novaseqcomb_R1.fastq.gz
PGT_PolII_HiCPmnt_Rep1_2Novaseqcomb_R2.fastq.gz
PGT_PolII_HiCPmnt_Rep2_2Novaseqcomb_R1.fastq.gz
PGT_PolII_HiCPmnt_Rep2_2Novaseqcomb_R2.fastq.gz
PH_PolII_HiCPmnt_Rep1_2Novaseqcomb_R1.fastq.gz
PH_PolII_HiCPmnt_Rep1_2Novaseqcomb_R2.fastq.gz
PH_PolII_HiCPmnt_Rep2_2Novaseqcomb_R1.fastq.gz
PH_PolII_HiCPmnt_Rep2_2Novaseqcomb_R2.fastq.gz
PP_PolII_HiCPmnt_Rep1_2Novaseqcomb_R1.fastq.gz
PP_PolII_HiCPmnt_Rep1_2Novaseqcomb_R2.fastq.gz
PP_PolII_HiCPmnt_Rep2_2Novaseqcomb_R1.fastq.gz
PP_PolII_HiCPmnt_Rep2_2Novaseqcomb_R2.fastq.gz
H9_S2P_HiCPmnt_Rep1_R1.fastq.gz

H9_S2P_HiCPmnt_Rep1_R2.fastq.gz
BETA_SA1_HiCPmnt_Rep1_2lanescomb_R1.fastq.gz
BETA_SA1_HiCPmnt_Rep1_2lanescomb_R2.fastq.gz
BETA_SA1_HiCPmnt_Rep2_2lanescomb_R1.fastq.gz
BETA_SA1_HiCPmnt_Rep2_2lanescomb_R2.fastq.gz
DE_SA1_HiCPmnt_Rep1_2lanescomb_R1.fastq.gz
DE_SA1_HiCPmnt_Rep1_2lanescomb_R2.fastq.gz
H9_SA1_HiCPmnt_Rep1_2lanescomb_R1.fastq.gz
H9_SA1_HiCPmnt_Rep1_2lanescomb_R2.fastq.gz
H9_SA1_HiCPmnt_Rep2_2lanescomb_R1.fastq.gz
H9_SA1_HiCPmnt_Rep2_2lanescomb_R2.fastq.gz
PGT_SA1_HiCPmnt_Rep1_2lanescomb_R1.fastq.gz
PGT_SA1_HiCPmnt_Rep1_2lanescomb_R2.fastq.gz
PGT_SA1_HiCPmnt_Rep2_2lanescomb_R1.fastq.gz
PGT_SA1_HiCPmnt_Rep2_2lanescomb_R2.fastq.gz
PH_SA1_HiCPmnt_Rep1_2lanescomb_R1.fastq.gz
PH_SA1_HiCPmnt_Rep1_2lanescomb_R2.fastq.gz
PH_SA1_HiCPmnt_Rep2_2lanescomb_R1.fastq.gz
PH_SA1_HiCPmnt_Rep2_2lanescomb_R2.fastq.gz
PP_SA1_HiCPmnt_Rep1_2lanescomb_R1.fastq.gz
PP_SA1_HiCPmnt_Rep1_2lanescomb_R2.fastq.gz
PP_SA1_HiCPmnt_Rep2_2lanescomb_R1.fastq.gz
PP_SA1_HiCPmnt_Rep2_2lanescomb_R2.fastq.gz
BETA_SA2_HiCPmnt_Rep1_2lanescomb_R1.fastq.gz
BETA_SA2_HiCPmnt_Rep1_2lanescomb_R2.fastq.gz
BETA_SA2_HiCPmnt_Rep2_2lanescomb_R1.fastq.gz
BETA_SA2_HiCPmnt_Rep2_2lanescomb_R2.fastq.gz
DE_SA2_HiCPmnt_Rep1_2lanescomb_R1.fastq.gz
DE_SA2_HiCPmnt_Rep1_2lanescomb_R2.fastq.gz
H9_SA2_HiCPmnt_Rep1_2lanescomb_R1.fastq.gz
H9_SA2_HiCPmnt_Rep1_2lanescomb_R2.fastq.gz
H9_SA2_HiCPmnt_Rep2_2lanescomb_R1.fastq.gz
H9_SA2_HiCPmnt_Rep2_2lanescomb_R2.fastq.gz
PGT_SA2_HiCPmnt_Rep1_2lanescomb_R1.fastq.gz
PGT_SA2_HiCPmnt_Rep1_2lanescomb_R2.fastq.gz
PGT_SA2_HiCPmnt_Rep2_2lanescomb_R1.fastq.gz
PGT_SA2_HiCPmnt_Rep2_2lanescomb_R2.fastq.gz
PH_SA2_HiCPmnt_Rep1_2lanescomb_R1.fastq.gz
PH_SA2_HiCPmnt_Rep1_2lanescomb_R2.fastq.gz
PH_SA2_HiCPmnt_Rep2_2lanescomb_R1.fastq.gz
PH_SA2_HiCPmnt_Rep2_2lanescomb_R2.fastq.gz
PP_SA2_HiCPmnt_Rep1_2lanescomb_R1.fastq.gz
PP_SA2_HiCPmnt_Rep1_2lanescomb_R2.fastq.gz
PP_SA2_HiCPmnt_Rep2_2lanescomb_R1.fastq.gz
PP_SA2_HiCPmnt_Rep2_2lanescomb_R2.fastq.gz
BETA_H3K27ac_DSG_MiCP_Rep1_2lanescomb_R1.fastq.gz
BETA_H3K27ac_DSG_MiCP_Rep1_2lanescomb_R2.fastq.gz
DE_H3K27ac_DSG_MiCP_Rep1_2lanescomb_R1.fastq.gz
DE_H3K27ac_DSG_MiCP_Rep1_2lanescomb_R2.fastq.gz
H9_H3K27ac_DSG_MiCP_Rep1_2lanescomb_R1.fastq.gz
H9_H3K27ac_DSG_MiCP_Rep1_2lanescomb_R2.fastq.gz
PGT_H3K27ac_DSG_MiCP_Rep1_2lanescomb_R1.fastq.gz
PGT_H3K27ac_DSG_MiCP_Rep1_2lanescomb_R2.fastq.gz
PP_H3K27ac_DSG_MiCP_Rep1_2lanescomb_R1.fastq.gz
PP_H3K27ac_DSG_MiCP_Rep1_2lanescomb_R2.fastq.gz
BETA_H3K4me1_DSG_MiCP_Rep1_2lanescomb_R1.fastq.gz
BETA_H3K4me1_DSG_MiCP_Rep1_2lanescomb_R2.fastq.gz
DE_H3K4me1_DSG_MiCP_Rep1_2lanescomb_R1.fastq.gz
DE_H3K4me1_DSG_MiCP_Rep1_2lanescomb_R2.fastq.gz
H9_H3K4me1_DSG_MiCP_Rep1_2lanescomb_R1.fastq.gz
H9_H3K4me1_DSG_MiCP_Rep1_2lanescomb_R2.fastq.gz
PGT_H3K4me1_DSG_MiCP_Rep1_2lanescomb_R1.fastq.gz
PGT_H3K4me1_DSG_MiCP_Rep1_2lanescomb_R2.fastq.gz
PP_H3K4me1_DSG_MiCP_Rep1_2lanescomb_R1.fastq.gz
PP_H3K4me1_DSG_MiCP_Rep1_2lanescomb_R2.fastq.gz
PGT_DpnII_HiC_Rep1_R1.fastq.gz
PGT_DpnII_HiC_Rep1_R2.fastq.gz
PP_DpnII_HiC_Rep2_R1.fastq.gz
PP_DpnII_HiC_Rep2_R2.fastq.gz
H9_DpnII_HiC_Rep2_R1.fastq.gz
H9_DpnII_HiC_Rep2_R2.fastq.gz
DE_DpnII_HiC_Rep2_R1.fastq.gz
DE_DpnII_HiC_Rep2_R2.fastq.gz
DE_DpnII_HiC_Rep1_R1.fastq.gz
PP_DpnII_HiC_Rep1_R1.fastq.gz
DE_DpnII_HiC_Rep1_R2.fastq.gz

BETA_DpnII_HiC_Rep1_R1.fastq.gz
PP_DpnII_HiC_Rep1_R2.fastq.gz
PH_DpnII_HiC_Rep1_R1.fastq.gz
PH_DpnII_HiC_Rep1_R2.fastq.gz
BETA_DpnII_HiC_Rep1_R2.fastq.gz
PGT_DpnII_HiC_Rep2_R1.fastq.gz
PGT_DpnII_HiC_Rep2_R2.fastq.gz
PH_DpnII_HiC_Rep2_R1.fastq.gz
BETA_DpnII_HiC_Rep2_R2.fastq.gz
BETA_DpnII_HiC_Rep2_R1.fastq.gz
PH_DpnII_HiC_Rep2_R2.fastq.gz
H9_DpnII_HiC_Rep1_R1.fastq.gz
H9_DpnII_HiC_Rep1_R2.fastq.gz
BETA_ATACRep5.normbyinput.qnorm_100bp.bw
BETA_Chd4_live_Cuttag.normbyinput.qnorm_100bp.bw
BETA_ChIPmentation_Input.scaletoCTCF.RPM.bw
BETA_cJun.normbyinput.qnorm_100bp.bw
BETA_CTCF.normbyinput.qnorm_100bp.bw
BETA_ESCO1.normbyinput.qnorm_100bp.bw
BETA_FOXA2.normbyinput.qnorm_100bp.bw
BETA_H3K9me2.normbyinput.qnorm_100bp.bw
BETA_H3K9me3.normbyinput.qnorm_100bp.bw
BETA_HDAC8.normbyinput.qnorm_100bp.bw
BETA_NIPBL.normbyinput.qnorm_100bp.bw
BETA_Rad21.normbyinput.qnorm_100bp.bw
BETA_RNASeq.2Reps.RPM.bw
BETA_S2P.normbyinput.qnorm_100bp.bw
BETA_WAPL.normbyinput.qnorm_100bp.bw
BETA_YY1.normbyinput.qnorm_100bp.bw
DE_ATACRep5.normbyinput.qnorm_100bp.bw
DE_Chd4_live_Cuttag.normbyinput.qnorm_100bp.bw
DE_ChIPmentation_Input.scaletoCTCF.RPM.bw
DE_cJun.normbyinput.qnorm_100bp.bw
DE_CTCF.normbyinput.qnorm_100bp.bw
DE_ESCO1.normbyinput.qnorm_100bp.bw
DE_FOXA2.normbyinput.qnorm_100bp.bw
DE_H3K9me2.normbyinput.qnorm_100bp.bw
DE_H3K9me3.normbyinput.qnorm_100bp.bw
DE_HDAC8.normbyinput.qnorm_100bp.bw
DE_NIPBL.normbyinput.qnorm_100bp.bw
DE_Rad21.normbyinput.qnorm_100bp.bw
DE_RNASeq.2Reps.RPM.bw
DE_S2P.normbyinput.qnorm_100bp.bw
DE_WAPL.normbyinput.qnorm_100bp.bw
DE_YY1.normbyinput.qnorm_100bp.bw
H9_Chd4_live_Cuttag.normbyinput.qnorm_100bp.bw
H9_ChIPmentation_Input.scaletoCTCF.RPM.bw
H9_cJun.normbyinput.qnorm_100bp.bw
H9_CTCF.normbyinput.qnorm_100bp.bw
H9_ESCO1.normbyinput.qnorm_100bp.bw
H9_H3K9me2.normbyinput.qnorm_100bp.bw
H9_H3K9me3.normbyinput.qnorm_100bp.bw
H9_HDAC8.normbyinput.qnorm_100bp.bw
H9_NIPBL.normbyinput.qnorm_100bp.bw
H9_Rad21.normbyinput.qnorm_100bp.bw
H9_RNASeq.2Reps.RPM.bw
H9_S2P.normbyinput.qnorm_100bp.bw
H9_WAPL.normbyinput.qnorm_100bp.bw
H9_YY1.normbyinput.qnorm_100bp.bw
PGT_ATACRep5.normbyinput.qnorm_100bp.bw
PGT_Chd4_live_Cuttag.normbyinput.qnorm_100bp.bw
PGT_ChIPmentation_Input.scaletoCTCF.RPM.bw
PGT_CTCF.normbyinput.qnorm_100bp.bw
PGT_ESCO1.normbyinput.qnorm_100bp.bw
PGT_FOXA2.normbyinput.qnorm_100bp.bw
PGT_H3K9me3.normbyinput.qnorm_100bp.bw
PGT_HDAC8.normbyinput.qnorm_100bp.bw
PGT_NIPBL.normbyinput.qnorm_100bp.bw
PGT_Rad21.normbyinput.qnorm_100bp.bw
PGT_RNASeq.2Reps.RPM.bw
PGT_S2P.normbyinput.qnorm_100bp.bw
PGT_WAPL.normbyinput.qnorm_100bp.bw
PGT_YY1.normbyinput.qnorm_100bp.bw
PH_Chd4_live_Cuttag.normbyinput.qnorm_100bp.bw
PH_ChIPmentation_Input.scaletoCTCF.RPM.bw
PH_cJun.normbyinput.qnorm_100bp.bw

PH_CTCF.normbyinput.qnorm_100bp.bw
PH_FOXA2.normbyinput.qnorm_100bp.bw
PH_H3K9me2.normbyinput.qnorm_100bp.bw
PH_H3K9me3.normbyinput.qnorm_100bp.bw
PH_NIPBL.normbyinput.qnorm_100bp.bw
PH_Rad21.normbyinput.qnorm_100bp.bw
PH_RNASeq.2Reps.RPM.bw
PH_S2P.normbyinput.qnorm_100bp.bw
PH_WAPL.normbyinput.qnorm_100bp.bw
PH_YY1.normbyinput.qnorm_100bp.bw
PP_ATACRep5.normbyinput.qnorm_100bp.bw
PP_Chd4_live_Cuttag.normbyinput.qnorm_100bp.bw
PP_ChIPmentation_Input.scaletoCTCF.RPM.bw
PP_CTCF.normbyinput.qnorm_100bp.bw
PP_ESCO1.normbyinput.qnorm_100bp.bw
PP_FOXA2.normbyinput.qnorm_100bp.bw
PP_H3K9me3.normbyinput.qnorm_100bp.bw
PP_HDAC8.normbyinput.qnorm_100bp.bw
PP_NIPBL.normbyinput.qnorm_100bp.bw
PP_Rad21.normbyinput.qnorm_100bp.bw
PP_RNASeq.2Reps.RPM.bw
PP_S2P.normbyinput.qnorm_100bp.bw
PP_WAPL.normbyinput.qnorm_100bp.bw
PP_YY1.normbyinput.qnorm_100bp.bw
BETA_H3K27ac_HiCPsg.normbyinput.qnorm_100bp.bw
BETA_H3K4me1_HiCPsg.normbyinput.qnorm_100bp.bw
DE_H3K27ac_HiCPsg.normbyinput.qnorm_100bp.bw
DE_H3K4me1_HiCPsg.normbyinput.qnorm_100bp.bw
H9_H3K27ac_HiCPsg.normbyinput.qnorm_100bp.bw
H9_H3K4me1_HiCPsg.normbyinput.qnorm_100bp.bw
PGT_H3K27ac_HiCPsg.normbyinput.qnorm_100bp.bw
PGT_H3K4me1_HiCPsg.normbyinput.qnorm_100bp.bw
PP_H3K27ac_HiCPsg.normbyinput.qnorm_100bp.bw
PP_H3K4me1_HiCPsg.normbyinput.qnorm_100bp.bw
BETA_ALL.ATAC.bowtie2.NFR115.p0.001_peaks.narrowPeak
BETA_Chd4_live_Cuttag.p0.00001_peaks.narrowPeak
BETA_cJun_CPmnt.p0.001_peaks.narrowPeak
BETA-CTCF-CPmnt-Rep2_ChIP.p0.00001_peaks.narrowPeak
BETA_ESCO1_CPmnt_peaks.narrowPeak
BETA_FOXA2_CPmnt.p0.00001_peaks.narrowPeak
BETA_H3K9me2_CPmnt.p0.00001_peaks.narrowPeak
BETA_H3K9me3_ChIPmentation_peaks.broadPeak
BETA_HDAC8_CPmnt_peaks.narrowPeak
BETA_NIPBL_CPmnt.p0.00001_peaks.narrowPeak
BETA_Rad21_ChIP_peaks.narrowPeak
BETA_S2P_ChIP_Rep_peaks.narrowPeak
BETA_WAPL_CPmnt.p0.00001_peaks.narrowPeak
BETA_YY1_CPmnt.p0.00001_peaks.narrowPeak
DE_ALL.ATAC.bowtie2.NFR115.p0.001_peaks.narrowPeak
DE_Chd4_live_Cuttag.p0.00001_peaks.narrowPeak
DE_cJun_CPmnt.p0.001_peaks.narrowPeak
DE-CTCF-CPmnt-Rep2_ChIP.p0.00001_peaks.narrowPeak
DE_ESCO1_CPmnt_peaks.narrowPeak
DE_FOXA2_CPmnt.p0.00001_peaks.narrowPeak
DE_H3K9me2_CPmnt.p0.00001_peaks.narrowPeak
DE_H3K9me3_ChIPmentation_peaks.broadPeak
DE_HDAC8_CPmnt_peaks.narrowPeak
DE_NIPBL_CPmnt.p0.00001_peaks.narrowPeak
DE_Rad21_ChIP_peaks.narrowPeak
DE_S2P_ChIP_peaks.narrowPeak
DE_WAPL_CPmnt.p0.00001_peaks.narrowPeak
DE_YY1_CPmnt.p0.00001_peaks.narrowPeak
H9_Chd4_live_Cuttag.p0.00001_peaks.narrowPeak
H9_cJun_CPmnt.p0.001_peaks.narrowPeak
H9-CTCF-CPmnt-Rep2_ChIP.p0.00001_peaks.narrowPeak
H9_DE_PGT_PP_PH_BETA.gene.meanTPM.genenamesorted
H9_ESCO1_CPmnt_peaks.narrowPeak
H9_H3K9me2_CPmnt.p0.00001_peaks.narrowPeak
H9_H3K9me3_ChIPmentation_peaks.broadPeak
H9_HDAC8_CPmnt_peaks.narrowPeak
H9_NIPBL_CPmnt.p0.00001_peaks.narrowPeak
H9_Rad21_ChIP_peaks.narrowPeak
H9_S2P_ChIP_peaks.narrowPeak
H9_WAPL_CPmnt.p0.00001_peaks.narrowPeak
H9_YY1_CPmnt.p0.00001_peaks.narrowPeak
PGT_ALL.ATAC.bowtie2.NFR115.p0.001_peaks.narrowPeak

PGT_Chd4_live_Cuttag.p0.00001_peaks.narrowPeak
PGT-CTCF-CPmnt-Rep2_ChIP.p0.00001_peaks.narrowPeak
PGT_ESCO1_CPmnt_peaks.narrowPeak
PGT_FOXA2_CPmnt.p0.00001_peaks.narrowPeak
PGT_H3K9me3_ChIPmentation_peaks.broadPeak
PGT_HDAC8_CPmnt_peaks.narrowPeak
PGT_NIPBL_CPmnt.p0.00001_peaks.narrowPeak
PGT_Rad21_ChIP_peaks.narrowPeak
PGT_S2P_ChIP_peaks.narrowPeak
PGT_WAPL_CPmnt.p0.00001_peaks.narrowPeak
PGT_YY1_CPmnt.p0.00001_peaks.narrowPeak
PH_Chd4_live_Cuttag.p0.00001_peaks.narrowPeak
PH_cJun_CPmnt.p0.001_peaks.narrowPeak
PH-CTCF-CPmnt-Rep2_ChIP.p0.00001_peaks.narrowPeak
PH_FOXA2_CPmnt.p0.00001_peaks.narrowPeak
PH_H3K9me2_CPmnt.p0.00001_peaks.narrowPeak
PH_H3K9me3_ChIPmentation_peaks.broadPeak
PH_NIPBL_CPmnt.p0.00001_peaks.narrowPeak
PH_Rad21_ChIP_peaks.narrowPeak
PH_S2P_ChIP_Rep_peaks.narrowPeak
PH_WAPL_CPmnt.p0.00001_peaks.narrowPeak
PH_YY1_CPmnt.p0.00001_peaks.narrowPeak
PP_ALL.ATAC.bowtie2.NFR115.p0.001_peaks.narrowPeak
PP_Chd4_live_Cuttag.p0.00001_peaks.narrowPeak
PP-CTCF-CPmnt-Rep2_ChIP.p0.00001_peaks.narrowPeak
PP_ESCO1_CPmnt_peaks.narrowPeak
PP_FOXA2_CPmnt.p0.00001_peaks.narrowPeak
PP_H3K9me3_ChIPmentation_peaks.broadPeak
PP_HDAC8_CPmnt_peaks.narrowPeak
PP_NIPBL_CPmnt.p0.00001_peaks.narrowPeak
PP_Rad21_ChIP_peaks.narrowPeak
PP_S2P_ChIP_peaks.narrowPeak
PP_WAPL_CPmnt.p0.00001_peaks.narrowPeak
PP_YY1_CPmnt.p0.00001_peaks.narrowPeak
BETA_H3K27ac_DSG_MiCP.2reps.allValidPairs.shuf11316134.hic
BETA_H3K27ac_HiCPmnt.2reps.allValidPairs.shuf14276425.hic
BETA_H3K4me1_DSG_MiCP.2reps.allValidPairs.shuf12905803.hic
BETA_H3K4me1_HiCPmnt.2reps.allValidPairs.shuf17251338.hic
BETA_H3K9me3_HiCPmnt_reps.shuf10534954.allValidPairs.hic
BETA_PolII_HiCPmnt.2reps.allValidPairs.shuf7487195.hic
BETA_SA1_HiCPmnt.2reps.allValidPairs.shuf16728285.hic
BETA_SA2_HiCPmnt.2reps.allValidPairs.shuf13515407.hic
DE_H3K27ac_DSG_MiCP.2reps.allValidPairs.shuf11316134.hic
DE_H3K27ac_HiCPmnt.2reps.allValidPairs.shuf14276425.hic
DE_H3K4me1_DSG_MiCP.2reps.allValidPairs.shuf12905803.hic
DE_H3K4me1_HiCPmnt.2reps.allValidPairs.hic
DE_H3K9me3_HiCPmnt_reps.shuf10534954.allValidPairs.hic
DE_PolII_HiCPmnt.2reps.allValidPairs.shuf7487195.hic
DE_SA1_HiCPmnt.2reps.allValidPairs.hic
DE_SA2_HiCPmnt.2reps.allValidPairs.hic
H9_H3K27ac_DSG_MiCP.2reps.allValidPairs.shuf11316134.hic
H9_H3K27ac_HiCPmnt.2reps.allValidPairs.shuf14276425.hic
H9_H3K4me1_DSG_MiCP.2reps.allValidPairs.shuf12905803.hic
H9_H3K4me1_HiCPmnt.2reps.allValidPairs.shuf17251338.hic
H9_H3K9me3_HiCPmnt_reps.shuf10534954.allValidPairs.hic
H9_PolII_HiCPmnt.2reps.allValidPairs.shuf7487195.hic
H9_S2P_HiCPmnt.allValidPairs.hic
H9_SA1_HiCPmnt.2reps.allValidPairs.shuf16728285.hic
H9_SA2_HiCPmnt.2reps.allValidPairs.shuf13515407.hic
PGT_H3K27ac_DSG_MiCP.2reps.allValidPairs.shuf11316134.hic
PGT_H3K27ac_HiCPmnt.2reps.allValidPairs.shuf14276425.hic
PGT_H3K4me1_DSG_MiCP.2reps.allValidPairs.shuf12905803.hic
PGT_H3K4me1_HiCPmnt.2reps.allValidPairs.shuf17251338.hic
PGT_H3K9me3_HiCPmnt_reps.shuf10534954.allValidPairs.hic
PGT_PolII_HiCPmnt.2reps.allValidPairs.shuf7487195.hic
PGT_SA1_HiCPmnt.2reps.allValidPairs.shuf16728285.hic
PGT_SA2_HiCPmnt.2reps.allValidPairs.shuf13515407.hic
PH_H3K27ac_HiCPmnt.2reps.allValidPairs.shuf14276425.hic
PH_H3K4me1_HiCPmnt.2reps.allValidPairs.shuf17251338.hic
PH_H3K9me3_HiCPmnt_reps.shuf10534954.allValidPairs.hic
PH_PolII_HiCPmnt.2reps.allValidPairs.shuf7487195.hic
PH_SA1_HiCPmnt.2reps.allValidPairs.shuf16728285.hic
PH_SA2_HiCPmnt.2reps.allValidPairs.shuf13515407.hic
PP_H3K27ac_DSG_MiCP.2reps.allValidPairs.shuf11316134.hic
PP_H3K27ac_HiCPmnt.2reps.allValidPairs.shuf14276425.hic
PP_H3K4me1_DSG_MiCP.2reps.allValidPairs.shuf12905803.hic

PP_H3K4me1_HiCPmnt.2reps.allValidPairs.shuf17251338.hic
PP_H3K9me3_HiCPmnt_reps.shuf10534954.allValidPairs.hic
PP_PolII_HiCPmnt.2reps.allValidPairs.shuf7487195.hic
PP_SA1_HiCPmnt.2reps.allValidPairs.shuf16728285.hic
PP_SA2_HiCPmnt.2reps.allValidPairs.shuf13515407.hic
PGT.rep2rep3_allValidPairs.shuf522269193.hic
PGT.rep2rep3_allValidPairs.shuf522269193.hic
PP.rep2rep3_allValidPairs.shuf522269193.hic
PP.rep2rep3_allValidPairs.shuf522269193.hic
H9.rep2rep3_allValidPairs.shuf522269193.hic
H9.rep2rep3_allValidPairs.shuf522269193.hic
DE.rep2rep3_allValidPairs.shuf522269193.hic
DE.rep2rep3_allValidPairs.shuf522269193.hic
BETA.rep1rep2_allValidPairs.shuf522269193.hic
BETA.rep1rep2_allValidPairs.shuf522269193.hic
PH.rep1rep2_allValidPairs.shuf522269193.hic
PH.rep1rep2_allValidPairs.shuf522269193.hic

Genome browser session
(e.g. UCSC)

http://genome.ucsc.edu/cgi-bin/hgTracks?
db=hg38&lastVirtModeType=default&lastVirtModeExtraState=&virtModeType=default&virtMode=0&nonVirtPosition=&position=chr2%3A25160915%2D25168903&hgsid=1611126427_mdEECIfeMN6Bjn7vycydZHmAcY3A

Methodology

Replicates

We performed 2 biological replicates for ChIP-Seq, CUT&Tag, RNA-Seq, ATAC-Seq, HiChIP, MiChIP and in-situ Hi-C, 3 biological replicates for immunohistochemistry, 3 biological replicates for RT-qPCR.

Sequencing depth

All replicates of ChIP-Seq and CUT&Tag are sequenced to a depth > 20 millions as paired-end reads.
All replicates of RNA-Seq, ATAC-Seq, HiChIP and MiChIP are sequenced to a depth > 30 millions as paired-end reads.
All replicates of in-situ Hi-C are sequenced to a depth > 600 millions as paired-end reads. Number of uniquely mapped paired-end reads in final Hi-C contacts are at a depth > 250 millions as reported in Table S1 provided in the manuscript.
H9_DpnII_HiC_Rep1: sequenced read pairs: 800,844,763; Hi-C contacts: 301,437,969
H9_DpnII_HiC_Rep2: sequenced read pairs: 1,415,278,918; Hi-C contacts: 478,788,858
DE_DpnII_HiC_Rep1: sequenced read pairs: 883,715,400; Hi-C contacts: 349,067,583
DE_DpnII_HiC_Rep2: sequenced read pairs: 911,847,758; Hi-C contacts: 346,593,333
PGT_DpnII_HiC_Rep1: sequenced read pairs: 1,050,548,494; Hi-C contacts: 511,827,226
PGT_DpnII_HiC_Rep2: sequenced read pairs: 617,775,928; Hi-C contacts: 266,693,868
PP_DpnII_HiC_Rep1: sequenced read pairs: 720,077,109; Hi-C contacts: 316,617,905
PP_DpnII_HiC_Rep2: sequenced read pairs: 964,731,174; Hi-C contacts: 403,354,104
PH_DpnII_HiC_Rep1: sequenced read pairs: 1,011,639,312; Hi-C contacts: 421,448,937
PH_DpnII_HiC_Rep2: sequenced read pairs: 1,187,716,185; Hi-C contacts: 490,408,013
BETA_DpnII_HiC_Rep1: sequenced read pairs: 1,008,888,448; Hi-C contacts: 408,196,266
BETA_DpnII_HiC_Rep2: sequenced read pairs: 1,152,357,509; Hi-C contacts: 457,601,167

Antibodies

Anti-RAD21 Abcam ab992
Anti-CTCF Abcam ab70303
Anti-YY1 Abcam ab109237
Anti-WAPL BETHYL A300-268A
Anti-NIPBL BETHYL A301-779A
Anti-cJun Abcam ab31419
Anti-RNAPII RPB1 Biolegend 920102
Anti-RNA polymerase II CTD repeat YSPTSPS (phospho S2) Abcam ab5095
Anti-SA1 BETHYL A302-579A
Anti-SA2 BETHYL A302-580A
Anti-CHD4 Abcam ab72418
Anti-FOXA2 Abcam ab256493
Anti-ESCO1 Sigma SAB1408225
Anti-H3K4me1 Abcam ab8895
Anti-H3K9me3 Abcam ab8898
Anti-H3K27ac Abcam ab4729
Anti-H3K9me2 Abcam ab1220
Anti-HDAC8 Abcam ab190954

Peak calling parameters

All reads were mapped to unique genomic regions using Bowtie2 (v2.3.5.1) and the hg38 human genome release. PCR duplicates were removed manually by Samtools (v1.9). Bedtools (v2.27.1) genome Coverage function was used to derive bedgraph files for further analysis. To compare changes in ChIP-seq signals, libraries were normalized by random picking to obtain the same numbers of reads. MACS2 (v2.1.2) was used to call peaks using default parameters with IgG ChIP-seq data as control.

Data quality

Raw reads qualities were assessed by FASTQC prior to procession. Peaks were called by using input as controls (see methods for details) with default parameters in MACS2.

Software

Juicer (Durand et al., 2016) https://github.com/theaidenlab/juicer/wiki
HiC-Pro (Servant, N., et al.， 2015) https://github.com/nservant/HiC-Pro
Juicebox (Durand et al., 2016) http://aidenlab.org/juicebox/

MACS2.0 (Liu, 2014)https://github.com/taoliu/MACS
Bowtie2 (Langmead, 2012) https://bowtie-bio.sourceforge.net/bowtie2/index.shtml
HISAT2 (Kim, D et al., 2019) https://github.com/DaehwanKimLab/hisat2
StringTie (Pertea, M et al., 2015) https://ccb.jhu.edu/software/stringtie/
Bismark (Krueger F, 2011) http://felixkrueger.github.io/Bismark/Docs/
SIP (Rowley MJ et al., 2020a) https://github.com/PouletAxel/SIP
SIPMeta (Rowley MJ et al., 2020b) https://github.com/PouletAxel/SIPMeta
Samtools (Li et al., 2009) https://sourceforge.net/projects/samtools/files/
Picard Tools http://picard.sourceforge.net; https://broadinstitute.github.io/picard/
TOPHAT2 (Kim et al., 2013) https://github.com/infphilo/tophat
Cufflinks (Trapnell et al., 2010) http://cole-trapnell-lab.github.io/cufflinks
DANPOS (Chen et al., 2013) https://sites.google.com/site/danposdoc/install
bedtools (Quinlan, 2014) http://bedtools.readthedocs.io/en/latest/
EdgeR （Robinson et al., 2010） https://bioconductor.org/packages/release/bioc/html/edgeR.html/
MAnorm (Shao et al., 2012) http://bcb.dfci.harvard.edu/~gcyuan/MAnorm/MAnorm.htm
Wellington-bootstrap (Piper et al., 2013) http://pythonhosted.org/pyDNase/
Java treeview https://sourceforge.net/projects/jtreeview/files/
Cluster3 http://bonsai.hgc.jp/~mdehoon/software/cluster/software.htm
FIMO (Grant et al., 2011) http://meme-suite.org/
MEME (Bailey et al., 2006) http://meme-suite.org/
FitHiChIP (Bhattacharyya, S al., 2019) https://ay-lab.github.io/FitHiChIP/
Fit-Hi-C (Ay et al., 2014) https://noble.gs.washington.edu/proj/fit-hi-c/

Flow Cytometry

Plots

Confirm that:

☒ The axis labels state the marker and fluorochrome used (e.g. CD4-FITC).

☒ The axis scales are clearly visible. Include numbers along axes only for bottom left plot of group (a 'group' is an analysis of identical markers).

☒ All plots are contour plots with outliers or pseudocolor plots.

☒ A numerical value for number of cells or percentage (with statistics) is provided.

Methodology

Sample preparation	Cultured spheroids were dissociated to single cells using Accutase (Thermol, A1110501) and fixed with 4% paraformaldehyde in PBS for 30 min at room temperature before intracellular flow cytometry. Fixed cells were permeabilized and blocked in PBS with 5% donkey serum (Jackson Immunoresearch) and 0.15% Triton X-100 (Sigma) for 20 min at room temperature. Cells were then stained with primary antibodies diluted with blocking buffer at 4°C overnight. After staining, cells were washed, incubated with appropriate secondary antibodies for 30 min at room temperature and then resuspended in FACS buffer for flow acquisition and analysis. Cells were filtered through a 40 micron nylon mesh (BD Biosciences) and loaded on a FACScanto (BD Biosciences) for flow cytometry analysis using FlowJo software (TreeStar). Antibodies used for intracellular flow cytometry are C-peptide (DSHB; GN-ID4) and Glucagon (R&D; MAB1249-SP), Chicken anti-Mouse IgG (H+L) Cross-Adsorbed Secondary Antibody (Alexa Fluor 594), and Goat Anti-Rat IgG H&L (Alexa Fluor 488).
Instrument	For analysis, FACScanto (BD Biosciences) flow cytometry was used.
Software	Data were collected using software for the FACScanto machine. Data was analyzed using FlowJo (v10.3).
Cell population abundance	Cells were dissociated to single cells using Accutase, washed once by culture media, and resuspended into 300ul of FACS buffer. Measure 1000 events at 300 events/sec.
Gating strategy	We gated the main population on the FSC/SSC plot by excluding the apparent populations of cell debris and cell doublets.

☐ Tick this box to confirm that a figure exemplifying the gating strategy is provided in the Supplementary Information.
